# Trabecular and cortical bone are unaltered in response to chronic lipopolysaccharide exposure via osmotic pumps in male and female CD-1 mice

**DOI:** 10.1371/journal.pone.0243933

**Published:** 2021-02-05

**Authors:** Kirsten N. Bott, Jenalyn L. Yumol, Elena M. Comelli, Panagiota Klentrou, Sandra J. Peters, Wendy E. Ward

**Affiliations:** 1 Department of Kinesiology, Brock University, St. Catharines, ON, Canada; 2 Department of Nutritional Sciences, University of Toronto, Toronto, ON, Canada; 3 Joannah and Brian Lawson Centre for Child Nutrition, University of Toronto, Toronto, ON, Canada; 4 Centre for Bone and Muscle Health, Brock University, St. Catharines, ON, Canada; 5 Department of Health Sciences, Brock University, St. Catharines, ON, Canada; Oklahoma State University, UNITED STATES

## Abstract

Chronic low-grade inflammation has been identified as an underlying cause of many diseases including osteoporosis. Lipopolysaccharide (LPS) is a potent inducer of the inflammatory response that can negatively affect bone outcomes by upregulating bone resorption and inhibiting bone formation. The objective of this study was to assess the longitudinal response of trabecular and cortical bone structure and bone mineral density to LPS continuously administered for 12 weeks in male and female CD-1 mice. Mice were assigned to one of four LPS groups at 8-weeks of age: placebo (0.0 μg/d), low (0.9 μg/d), mid (3.6 μg/d) and high (14.4 μg/d) dose. Trabecular and cortical bone outcomes were measured at 8, 12, 16, and 20 weeks of age using *in vivo* micro-computed tomography. The anticipated serum LPS dose-dependent response was not observed. Therefore, the low, mid, and high LPS groups were combined for analysis. Compared to the placebo group, endpoint serum LPS was elevated in both males (*p* < 0.05) and females (*p* < 0.05) when all LPS treatment groups were combined. However, there was no significant change in trabecular or cortical bone outcomes in the combined LPS groups compared to the placebo following the 12-week LPS intervention for either sex. This suggests that although serum LPS was elevated following the 12-week LPS intervention, the dosages administered using the osmotic pumps was not sufficient to negatively impact trabecular or cortical bone outcomes in either male or female CD-1 mice.

## Introduction

Inflammation is an important response to injury or infection that signals the immune system to repair damaged tissues or protect the body from foreign invaders. Whereas, chronic low-grade inflammation is often characterized by low concentrations of circulating pro-inflammatory cytokines [1]. Chronic low-grade inflammation has been implicated in the pathogenesis of a number of diseases in both humans and animals including, inflammatory bowel disease [2], pancreatitis [3], rheumatoid arthritis [4], non-alcoholic fatty liver disease [5], and cardiovascular disease [6]—all of which have been associated with low bone mineral density (BMD). Bone is continuously remodeled throughout the life span, mediated by the activity of bone forming osteoblasts and bone resorbing osteoclasts [7]. However, in the presence of elevated pro-inflammatory cytokines, the balance between osteoblast and osteoclast activity becomes dysregulated in favor of bone resorption. Specifically, interleukin (IL) 1-β, IL-6, and tumor necrosis factor-α (TNF-α) are associated with chronic low-grade inflammation [8] and increased bone resorption by upregulating osteoclastogenesis, preventing osteoclast apoptosis, and inhibiting osteoblastogenesis [9]. Upregulated bone resorption leads to lower bone mass and increases the risk of fragility fractures. Fragility fractures are associated with high rates of morbidity and mortality and are a public health concern as they are costly and reduce an individual’s quality of life [10]. Since chronic low-grade inflammation has been identified as a cause for exacerbated bone loss it is important to develop models to investigate potential prevention or treatment strategies.

Lipopolysaccharide (LPS) is a component of the outer membrane of Gram-negative bacteria such as those found in the gut microbiota (e.g. *Bacteroides*, *Prevotella*, *Escherichia*). LPS can be released from these bacteria, with limited amounts passing through the gut epithelium into circulation under normal conditions. However, with increased intestinal permeability, which has been associated with obesity and even aging [11], there is an elevation in circulating LPS that can stimulate chronic low-grade inflammation. LPS can also directly alter bone metabolism through its interaction with toll-like receptors (TLRs) that activate various signaling pathways to favour bone resorption [12]. These TLRs link the immune and skeletal systems by detecting pathogens; specifically, TLR4, which is expressed on the membranes of osteoblasts, osteoclasts, and macrophages, binds circulating LPS [12]. Previously, slow-release pellets containing LPS have been subcutaneously implanted to induce chronic low-grade inflammation with a focus on bone outcomes in both mice [13–15] and rats [16–18]. These studies demonstrated that LPS treatment negatively impacted trabecular bone structure, BMD, and bone strength in both males [14,16] and females [13,15,17,18]. However, only endpoint bone outcomes were analyzed and, in some cases, LPS treatment was for a relatively short duration; longitudinal measurement of bone outcomes is important for identifying key timepoints for developing intervention strategies. Moreover, although Droke et al. [13] quantified the lymphocytes to neutrophil ratio to assess the inflammatory state, serum LPS was not measured in any of the studies; analyzing serum LPS provides information on the release rate of the employed delivery system.

Slow-release pellets–not containing LPS—have been reported to have inconsistent release rates [19–21], but studies using slow-release pellets to deliver LPS have observed an inflammatory effect [13–18]. Whether a controlled and consistent dose of LPS stimulates a similar inflammatory effect while possibly more closely mimicking the scenario in humans, remains to be determined [22]. Previous studies using slow-release 17β-estradiol pellets demonstrated that plasma estradiol, indicative of pellet release rate, either significantly fluctuated [19] or resulted in an initial extreme supraphysiological dose followed by a substantial decrease [20,21]. This suggests that pellets do not provide a consistent dose. Animal models using LPS to investigate this association should reflect this consistently elevated circulating LPS to induce chronic low-grade inflammation. An alternative to pellets for long-term administration with an established consistent release rate are osmotic pumps [23,24]. This system uses the principle of osmosis; once implanted, body water enters the salt layer through the semipermeable membrane, resulting in compression of the reservoir pushing out the solution through the exit port. The composition of the membrane determines solution release rate and delivery duration, which can range from weeks to months [25].

Although the link between LPS and its negative effects on bone metabolism via the suppression of osteoblast and upregulation of osteoclast activities is known [12], to date trabecular and cortical bone structure outcomes and BMD have not been tracked longitudinally. Repeatedly measuring outcomes within the same animal over time not only increases the power of the study design by serving as internal controls, but also allows for critical time points to be identified for potential interventions. The CD-1 mouse was selected since it is an outbred strain providing more heterogeneity to reflect variability among human population and mice represent an accelerated model for tracking bone outcomes longitudinally that allows for multiple *in vivo* μCT scans. The objective of this study was to assess the longitudinal response of trabecular and cortical bone structure and BMD to continuously administered LPS for a duration of 12 weeks in both male and female CD-1 mice. We hypothesized that male and female CD-1 mice implanted with osmotic pumps filled with LPS from 8 to 20 weeks of age would negatively affect bone structure and BMD over the time course of the study measured at 4-week intervals.

## Methods

### Animals

All procedures that involved animals were in compliance with the Canadian Council on Animal Care and approved by the Brock University Animal Care Committee (AUP #17-06-01). Both male and female 7-week old CD-1 mice (male weight 32.2±2.5 g, n = 42; female weight 27.1±1.6 g, n = 38) were ordered from Charles Rivers (Canada) and allowed to acclimatize for 5 days in the Comparative Bioscience Facility. Females were housed 4–5 per cage, while males were housed individually. All mice were kept on a standard 12-hour light: 12-hour dark cycle and had *ad libitum* access to food (AIN-93G diet (TD.94045), Harlan Teklad, Mississauga, ON) and water.

### Experimental design

Lipopolysaccharide (LPS; *Escherichia coli* Serotype O55:B5 by phenol extraction; Sigma, St. Louis, MO) was mixed in sterile PBS at a concentration of 4 μg/μL for the high-dose (14.4 μg/d) and serially diluted to 1 μg/μL for the mid-dose (3.6 μg/d) and 0.25μg/μL for the low-dose (0.9 μg/d). These concentrations were selected based on a previous study by Droke et al. that used slow-release pellets which delivered 0, 0.133, 1.33, and 13.3 μg LPS/d [13]. All LPS solutions were filtered through a PVDF 0.22 μm filter (Millex, Duluth, GA) then aliquoted and stored at -20°C until use. Mice were randomly assigned to one of four groups (n = 9-12/group): placebo (received only PBS; 0 μg/day), low-dose LPS (0.9 μg/day), mid-dose LPS (3.6 μg/day), or high-dose LPS (14.4 μg/day). To ensure the average and standard deviation for baseline body weight was not significantly different among groups and could potentially influence final results, the original allocation of a mouse to a specific group was changed.

The microcomputed tomography (μCT) scanning parameters and analysis have previously been published [26] for the male and female control group as they were a part of this larger study. Mice underwent *in vivo* scans of the hindlimb at 8, 12, 16, and 20 weeks of age by μCT. Pumps were filled the day prior to scans and primed in sterile PBS for subdermal implantation. Immediately following the scan performed at 8, 12 and 16 weeks of age, an Alzet osmotic pump (Durect Corporation, Cupertino, CA), with a release rate of 0.15 μL/hour designed to provide a consistent dose for 42 days (model 2006), was subcutaneously implanted in the dorsal region of the neck. This was for a total of three pump implantations over the course of the study and occurred immediately after a μCT scan while mice were still anesthetized. Metacam was subcutaneously injected to assist with any discomfort and bupivacaine was applied to the incision site as a nerve blocker. In short, an incision was made on the dorsal region of the neck, hemostat forceps were used to create a subcutaneous pocket, the pump was inserted, and the incision was closed. Initially, surgical glue was used to close the incisions but was switched to surgical staples to minimize the reopening of incisions. Mice were monitored daily to ensure proper incision healing. If an incision reopened, mice were anaesthetized, and the incision was closed with either surgical staples or sutures.

### Determination of trabecular structure and bone mineral density, and cortical bone structure and tissue mineral density in tibia

#### *In vivo* scanning using μCT

Scans were completed using μCT (SkyScan 1176, Bruker-microCT, Kontich, Belgium) and host software (1176 version 1.1, Bruker-microCT, Kontich, Belgium) as previously described [26–28]. To ensure consistent animal sedation during the *in vivo* scans isoflurane anesthetic was used since the flow rate could be tightly controlled and allowed mice to remain still throughout the scan, essential for obtaining a clear scan. In short, mice were anaesthetized in an induction chamber with isoflurane at a constant flow rate of 2–5% and transitioned to a nose cone housed inside the μCT for maintenance of the anesthetic. Ophthalmic gel was applied to the eyes to prevent dryness. Scans of the hindlimb were performed with the mouse positioned on its back with the scanning leg extended and non-scanning leg and tail secured away from scanning region, as previously described [27]. Scans were set to an isotropic resolution of 9 μm with a 1 mm aluminum filter and scanning parameters were specified at 3350 ms exposure time, 40 kV and 300 μA using a rotation step of 0.8° over 180° with no frame averaging [26] (Table 1). Scanning parameters are summarized in Table 1. To minimize any potential effect of scan order, one mouse from each group was scanned (placebo, low, mid, and high) and this order was subsequently repeated until each mouse had been scanned.

**Table 1:** Parameters used for μCT acquisition scans and reconstruction of images.

**μCT scanning parameters**	
Isotropic resolution	9 μm
Filter	1 mm aluminum
Voltage	40 kV
Current	300 μA
Rotation step	0.8°
Exposure time	3350 ms
**Reconstruction Parameters**	
Post-alignment compensations	+5/-5
Smoothing	4
Beam hardening	40%
Ring Artifact	6
Dynamic threshold	0.00000–0.09392

### Image processing and analysis

As previously described, GPURecon Server program (version 1.7.3.2, Bruker-microCT) and NRecon Reconstruction (version 1.7.3.1, Bruker-microCT) were used to reconstruct cross-sectional images [26]. The same reconstruction parameters were applied to all images for males and females: variable post-alignment compensations within +5/-5, smoothing (4), beam hardening (40%), ring artifact (6), and dynamic image range for the X-ray attenuation coefficient (0.00000–09392) (Table 1). Reconstruction parameters are summarized in Table 1. Images were reoriented using DataViewer (version 1.5.6.2, Bruker microCT), followed by region of interest (ROI) selection for trabecular and cortical bone using CTAnalyzer software (version 1.17.7.2+, Bruker-microCT). ROI selection was landmarked at the formation of the mineralized cartilage “bridge” where the primary spongiosa of the proximal tibia metaphysis disconnects. Using the landmarked transaxial slice, the top slice of the trabecular ROI was offset by 75 slices (0.66 mm) from the metaphyseal end of the growth plate. Trabecular bone was analyzed at the proximal tibia metaphysis with a 70 slice (0.62 mm) ROI extending distally towards the ankle and was manually contoured within the endocortical shell. Cortical bone was analyzed in the tibia metaphysis using an 850 slice (7.47 mm) offset from the landmarked transaxial slice with a 200 slice (1.76 mm) ROI extending distally towards the ankle. The cortical bone ROI was selected by excluding the marrow cavity by manually contouring a subtractive ROI, followed by manually contouring around the periosteal perimeter from the previously saved subtractive ROI. To separate trabecular and cortical bone from non-bone tissue, a global threshold of 65–255 and 105–255 was applied, respectively. Standard phantoms with known densities of 0.25 g/cm^3^ and 0.75 g/cm^3^ were used to calibrate the μCT for BMD analysis.

Proximal tibia trabecular bone outcomes assessed using 3D analysis included: bone volume fraction (BV/TV), trabecular thickness (Tb.Th), trabecular number (Tb.N), trabecular separation (Tb.Sp), degree of anisotropy (DA), structural model index (SMI), and volumetric BMD (vBMD) [29]. Cortical bone outcomes assessed using 2D analysis included: cortical area fraction (Ct.Ar/Tt.Ar), cortical thickness (Ct.Th), periosteal perimeter (Ps.Pm), endocortical perimeter (Ec.Pm), total area (Tt.Ar), medullary area (Ma.Ar), and tissue mineral density (TMD) [29] (CTAnalyzer, version 1.17.7.2+, Bruker-microCT).

### Serum LPS and leukocyte count

At the end of the study, mice were anesthetized, and blood was collected via cardiac puncture. Leukocytes were counted via manual microscopy of blood smears. Briefly, a small drop of blood was smeared across a slide and allowed to air dry. Following which 100 μL of the Wright stain (Harleco^®^, Millipore Sigma, St. Louis, MO) was added for two minutes and then mixed with 100 μL dH_2_O for one minute followed by an additional 100 μL of dH_2_O for two minutes. Excess liquid on the slide was discarded. The blood smears were viewed on a Leica HC microscope at 20x magnification. Because the amount of blood applied to a slide varied, we averaged the number of leukocytes per field of view–for a total of 10 fields of view—instead of quantifying the entire slide. Remaining blood was centrifuged to collect serum for determination of circulating LPS concentrations. Serum LPS was analyzed using a mouse LPS ELISA kit (Cusabio Technology LLC, Wuhan, China) according to manufacturer instructions. This kit has been used to analyze serum LPS in previous studies [30–32].

### Statistics

All values are presented as means ± standard error (SEM). For serum LPS a one-way ANOVA was used to compare all groups. Due to the overall similarity in serum LPS among LPS treatment groups, the low, mid, and high LPS groups were combined for analysis. A two-tailed unpaired t-test with Welch’s correction was used to compare LPS treatment to the placebo group for both the serum LPS and leukocyte count. Trabecular and cortical bone outcomes were analyzed using a two-way ANOVA (LPS treatment x time) with Bonferroni correction for multiple comparisons. All statistical analysis was done using GraphPad Prism (version 8.3.0) with statistical significance set to *p* ≤ 0.05.

## Results

### Serum LPS & leukocyte count

**Fig 1:**
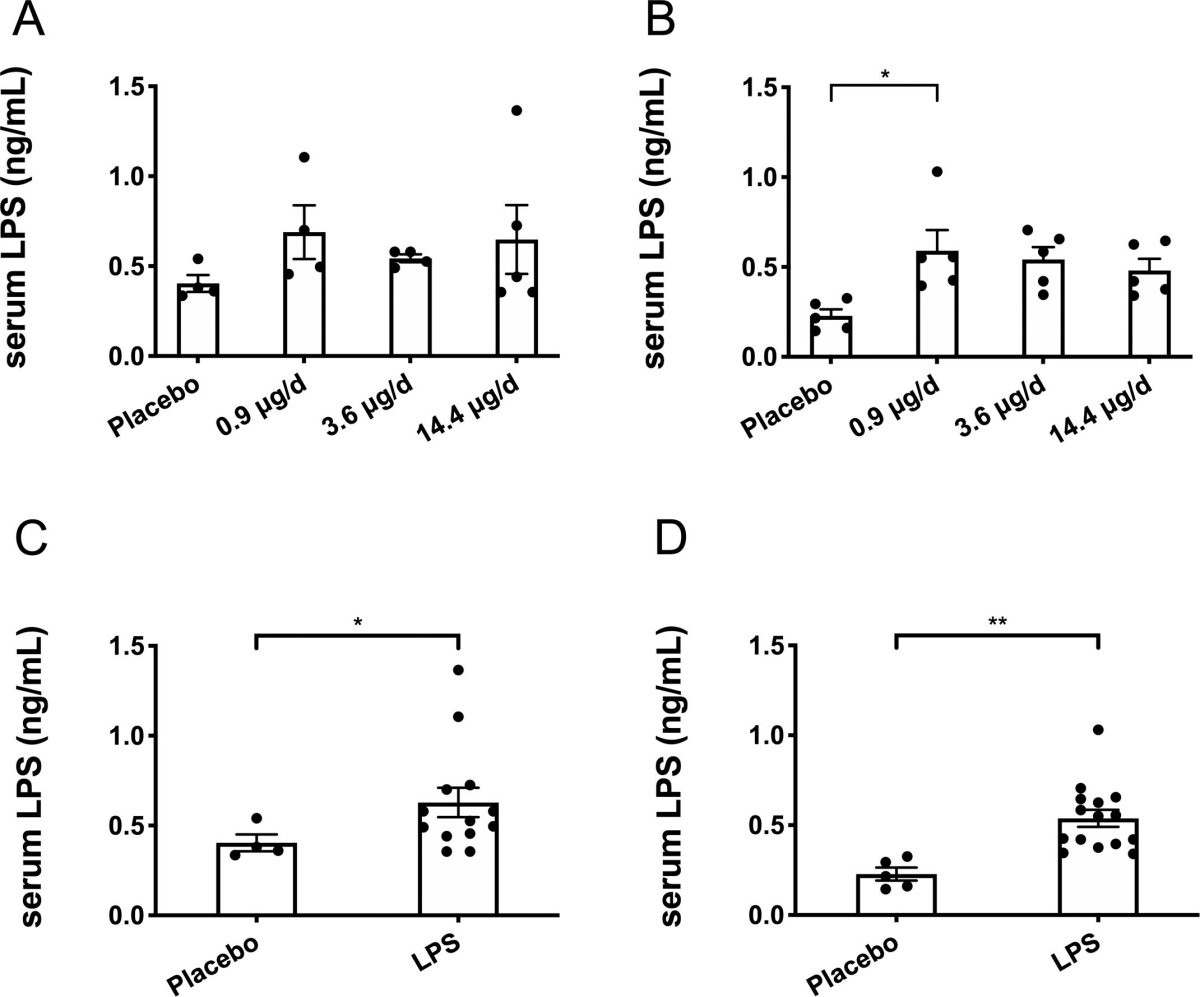
Endpoint serum lipopolysaccharide (LPS) concentrations. Serum LPS was unchanged among the different LPS groups (low 0.9 μg/d, mid 3.6 μg/d, and high 14.4 μg/d dosages), therefore, LPS groups were combined for analysis. (A) Male serum LPS was not different between any of the groups, placebo (n = 4), 0.9 μg/d (n = 4), 3.6 μg/d (n = 4), or 14.4 μg/d (n = 5) (*p* > 0.05). (B) Female serum LPS was elevated in the 0.9 μg/d group (n = 5) compared to the placebo group (n = 5) (*p* < 0.05), but did not differ between any of the other groups, 3.6 μg/d (n = 5) or 14.4 μg/d (n = 5). (C) When male groups receiving LPS were combined, serum LPS was increased in the LPS group (n = 13) compared to the placebo group (n = 4) (*p* < 0.05). (D) When female groups receiving LPS were combined, serum LPS was increased in the LPS group (n = 15) compared to the placebo group (n = 5) (*p* < 0.01). Data represented as mean ± SEM, statistical significance denoted as *p < 0.05, and **p < 0.01.

**Fig 2:**
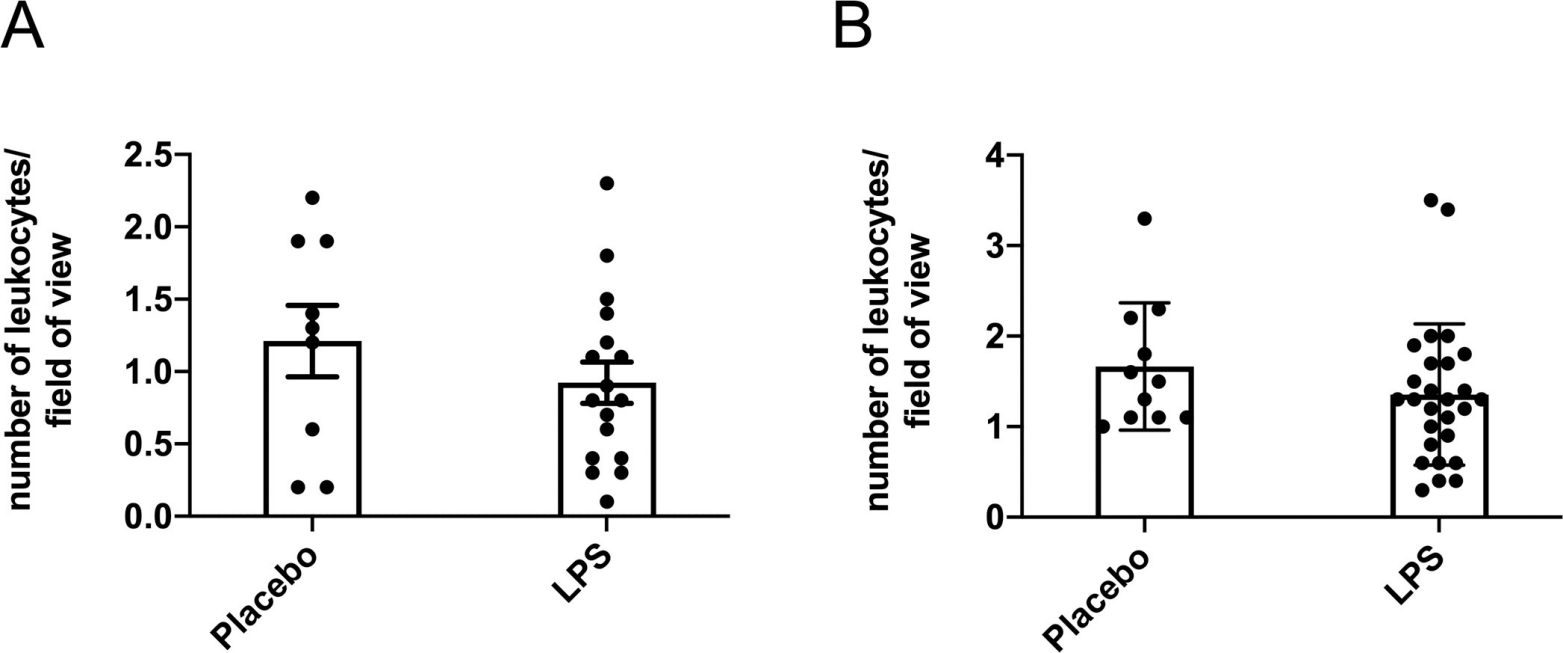
Blood smear leukocyte counts. Leukocytes counts were unchanged following 12-weeks of lipopolysaccharide (LPS) exposure in both (A) males (n = 9–17) (B) and females (n = 11–27). Ten fields of view per sample were analyzed, captured at 20x magnification.

**Fig 3:**
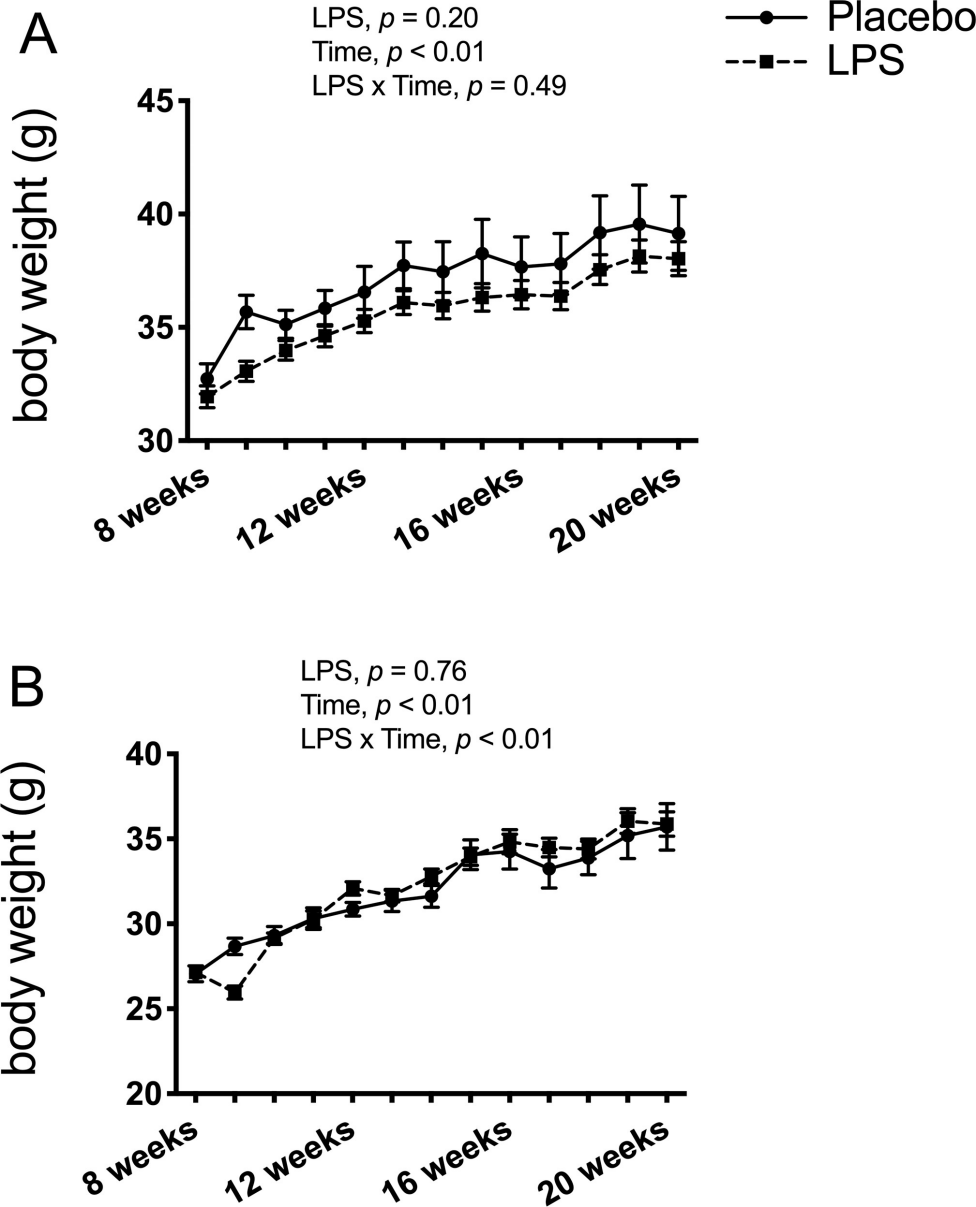
Βody weight. Lipopolysaccharide (LPS) treatment did not affect body weight. (A) Male body weight showed a significant main effect for time (*p* < 0.01) but no significant time x LPS interaction (*p* > 0.05), and no significant differences (*p* > 0.05 for LPS treatment effect) between the placebo (n = 12) and LPS group (n = 30). (B) Female body weight showed a significant time x LPS treatment interaction (*p* < 0.01) and main effect for time (*p* < 0.01), but with no significant differences (*p* > 0.05 for LPS treatment) between the placebo (n = 11) and LPS group (n = 27). Data represented as mean ± SEM.

Endpoint serum LPS concentrations did not differ between any of the treatment groups for the males (*p* > 0.05; Fig 1A) and only the low-dose group had higher serum LPS compared to the placebo group for the females (*p* < 0.05; Fig 1B) with none of the other groups differing. Thus, data from the three LPS dosages were combined to create one LPS group by sex. Here, LPS treatment for 12-weeks was found to significantly elevate circulating LPS by 36% in males (*p* < 0.05; Fig 1C) and by 58% in females (*p* < 0.01; Fig 1D) compared to placebo. Leukocyte counts were not statistically different between the placebo and LPS groups for both males (*p* > 0.05; Fig 2A) and females (*p* > 0.05; Fig 2B). Additionally, placebo and LPS groups similarly gained body weight throughout the 12-weeks for both male (Fig 3A) and female (Fig 3B) mice.

### Trabecular bone outcomes

**Fig 4:**
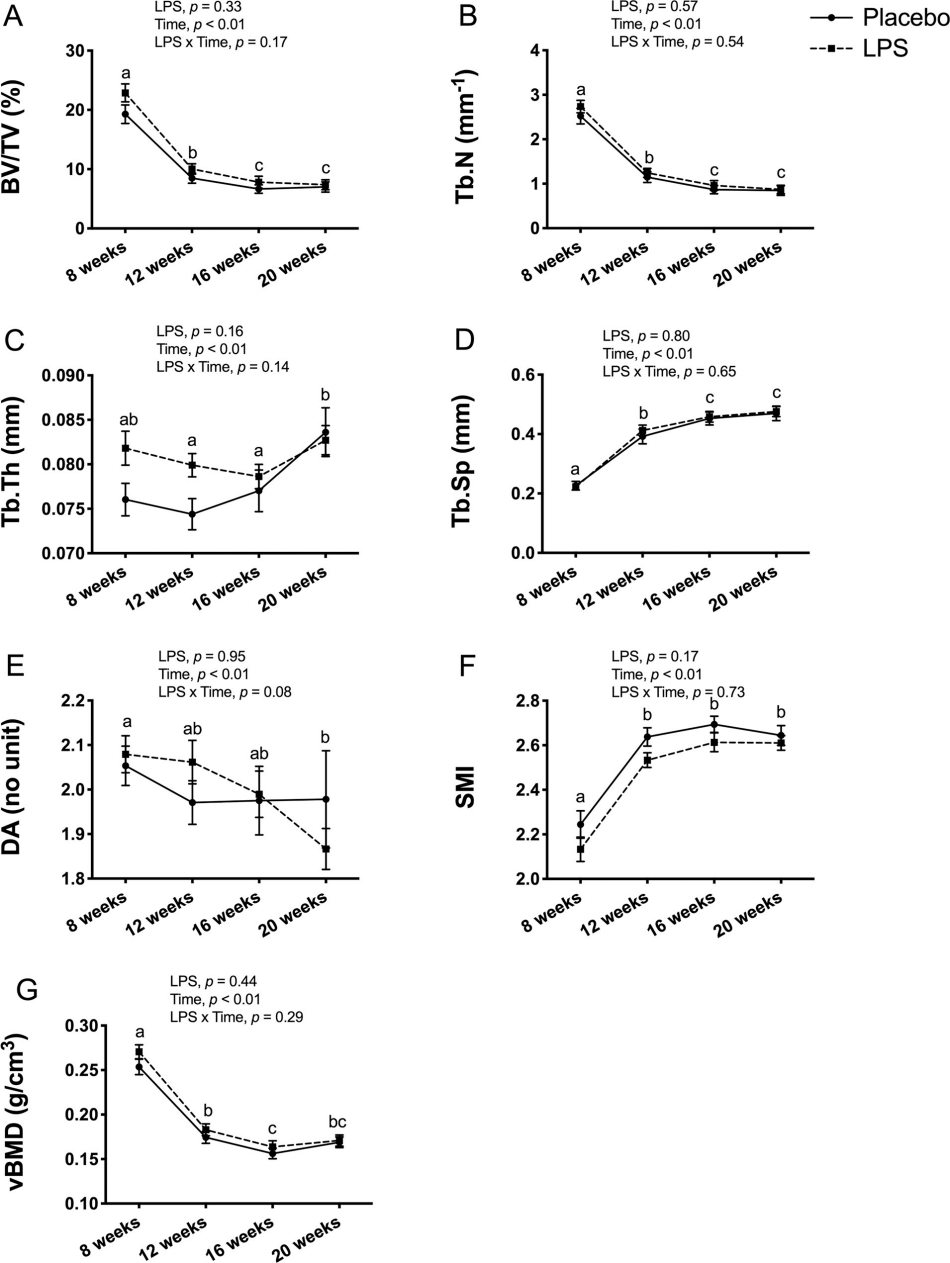
Male trabecular bone structure and volumetric bone mineral density (vBMD) at proximal tibia metaphysis. Trabecular bone structure and vBMD were unaltered with 12-weeks of lipopolysaccharide (LPS) treatment between the placebo (n = 12) and LPS group (n = 30). (A) Bone volume fraction (BV/TV) (B) Trabecular number (Tb.N) (C) Trabecular thickness (Tb.Th) (D) Trabecular separation (Tb.Sp) (E) Degree of anisotropy (DA) (F) Structural model index (SMI) (G) Trabecular vBMD (g/cm^3^). Data represented as mean±SEM. Significant differences between timepoints (main effect for time) are denoted with lowercase letters.

In male mice, trabecular bone outcomes at the proximal tibia metaphysis showed no significant LPS x time interaction for any of the outcomes (Fig 4). As expected, there was a main effect for time, which reflects an overall decrease from 8-weeks to 20-weeks of age for BV/TV (*p* < 0.01; Fig 4A), Tb.N (*p* < 0.01; Fig 4B), DA (*p* < 0.01; Fig 4E), and vBMD (*p* < 0.01; Fig 4G), and an increase for Tb.Th (*p* < 0.01; Fig 4C), Tb.Sp (*p* < 0.01; Fig 4D), and SMI (*p* < 0.01; Fig 4F).

**Fig 5:**
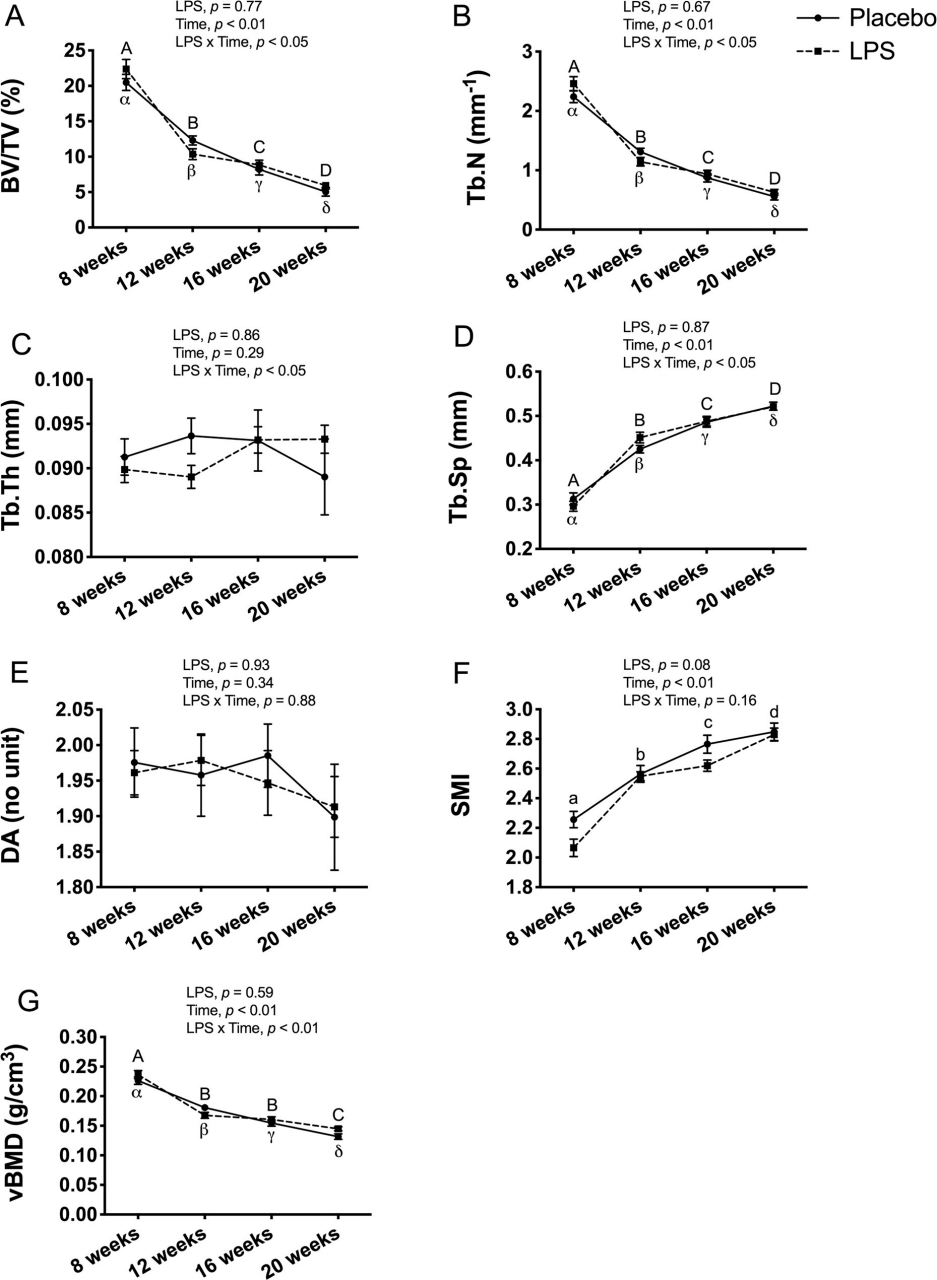
Female trabecular bone structure and volumetric bone mineral density (vBMD) at proximal tibia metaphysis. Trabecular bone structure and vBMD were unaltered with 12-weeks of lipopolysaccharide (LPS) treatment between the placebo (n = 11) and LPS group (n = 27). (A) Bone volume fraction (BV/TV) (B) Trabecular number (Tb.N) (C) Trabecular thickness (Tb.Th) (D) Trabecular separation (Tb.Sp) (E) Degree of anisotropy (DA) (F) Structural model index (SMI) (G) Trabecular vBMD (g/cm^3^). Data represented as mean±SEM. Significant differences between timepoints (main effect for time) are denoted with lowercase letters; Significant differences between timepoints within a group (interaction effect) are denoted with Greek letters for placebo group and uppercase letters for LPS group.

In female mice, there was a significant main effect for time and a significant LPS x time interaction for BV/TV (*p* < 0.05; Fig 5A), Tb.N (*p* < 0.05; Fig 5B), Tb.Sp (*p* < 0.05; Fig 5F), and vBMD (*p* < 0.01; Fig 5G). However, there was no effect for LPS treatment for any of these trabecular bone outcomes. Thus, further post-hoc analysis confirmed significant changes over time, with BV/TV (*p* < 0.01; Fig 5A), Tb.N (*p* < 0.01; Fig 5B), and vBMD (*p* < 0.01; Fig 5G) decreasing and Tb.Sp (*p* < 0.01; Fig 5D) increasing from 8-weeks to 20-weeks of age, but the differences between the LPS and placebo groups were not significant. A significant LPS x time interaction was also found for Tb.Th (*p* < 0.05; Fig 5D), but with no significant main effect for either time or LPS treatment. For DA, there was no significant LPS x time interaction and no main effect for either time or LPS (Fig 5E) reflecting that DA was unaltered with time in both groups. Likewise, there was no significant LPS x time interaction for SMI and no main effect for LPS, but the main effect for time was significant (*p* < 0.01; Fig 5F) reflecting that SMI (*p* < 0.01; Fig 5F) increased over time regardless of group.

### Cortical bone outcomes

**Fig 6:**
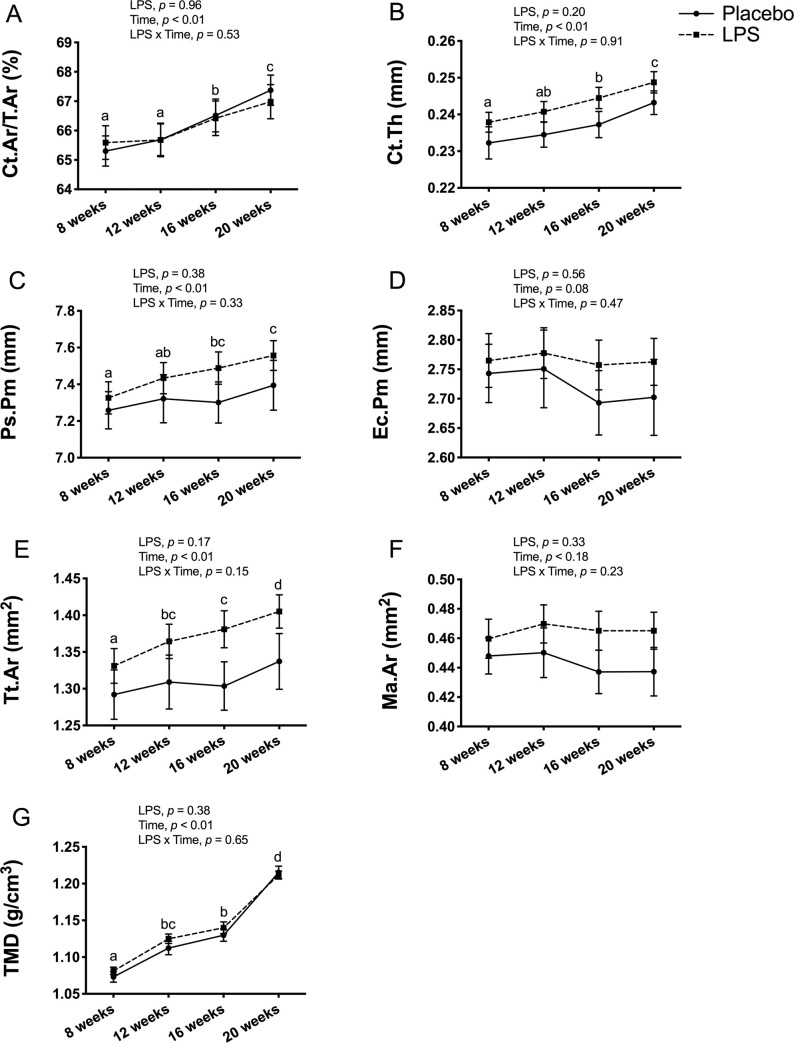
Male cortical bone structure and tissue mineral density (TMD) at tibia midpoint. Cortical bone outcomes and TMD were unaltered with 12-weeks of lipopolysaccharide (LPS) treatment between the placebo (n = 12) and LPS group (n = 30). (A) Cortical area fraction (Ct.Ar/Tt.Ar) (B) Cortical thickness (Ct.Th) (C) Periosteal perimeter (Ps.Pm) (D) Endocortical perimeter (Ec.Pm) (E) Total area (Tt.Ar) (F) Medullary area (Ma.Ar) (G) Cortical TMD (g/cm^3^). Data represented as mean±SEM. Significant differences between timepoints (main effect for time) are denoted with lowercase letters.

Cortical bone outcomes at the tibia midpoint had no LPS x time interaction for any of the outcomes in male mice (Fig 6). With the exception of Ec.Pm and Ma.Ar, all cortical bone outcomes had a significant main effect for time, but no significant main effect for LPS treatment. The main effect for time reflected an overall increase from 8-weeks of age to 20-weeks of age for Ct.Ar/Tt.Ar (*p* < 0.01; Fig 6A), Ct.Th (*p* < 0.01; Fig 6B), Ps.Pm (*p* < 0.01; Fig 6C), Tt.Ar (*p* < 0.01; Fig 6E), and TMD (*p* < 0.01; Fig 6G). In contrast, Ec.Pm (*p* > 0.05; Fig 6D) and Ma.Ar (*p* > 0.05; Fig 6F) were unchanged over time.

**Fig 7:**
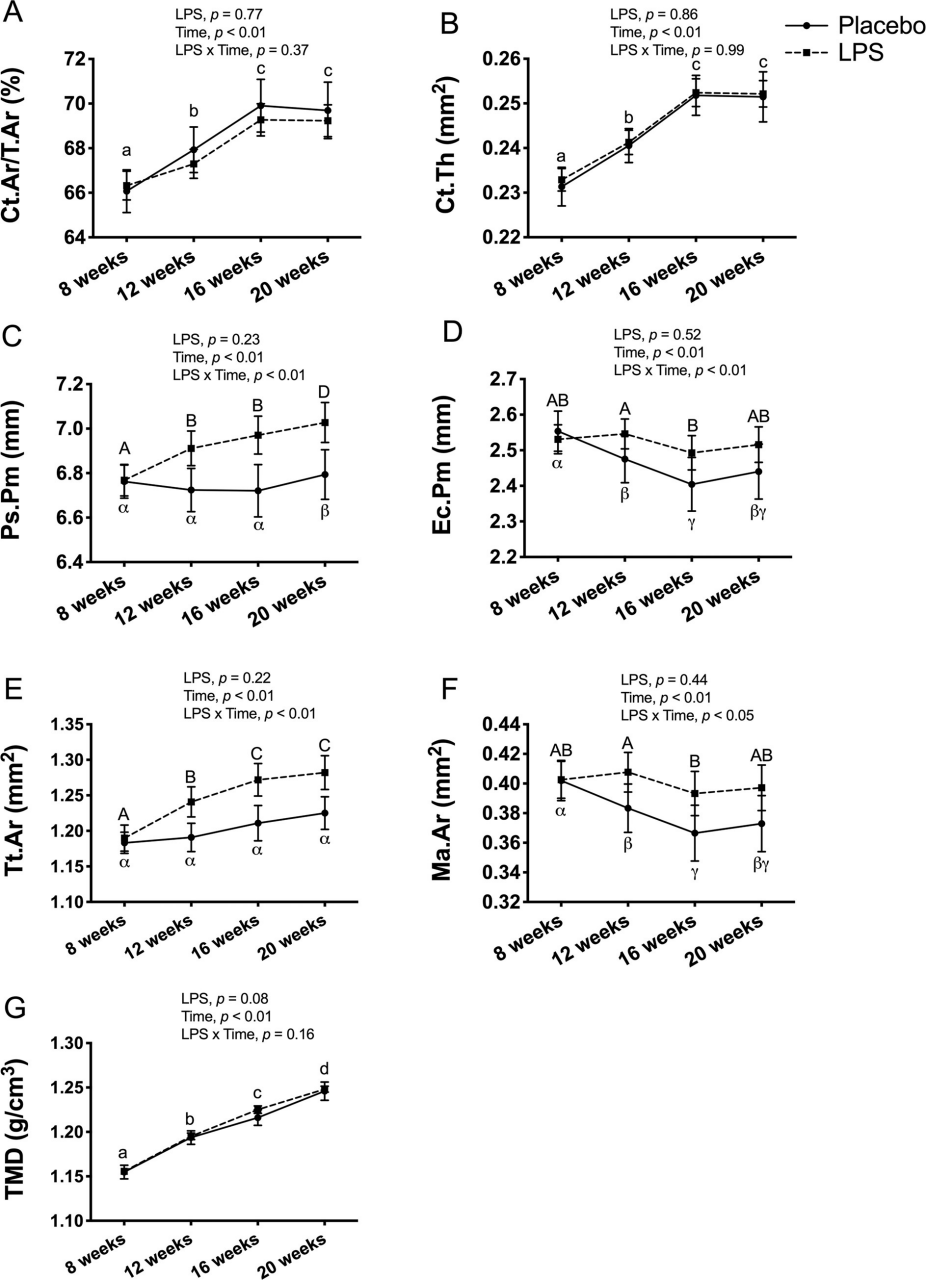
Female cortical bone structure and tissue mineral density (TMD) at tibia midpoint. Cortical bone outcomes and TMD were unaltered with 12-weeks of lipopolysaccharide (LPS) treatment between the placebo (n = 11) and LPS group (n = 27). (A) Cortical area fraction (Ct.Ar/Tt.Ar) (B) Cortical thickness (Ct.Th) (C) Periosteal perimeter (Ps.Pm) (D) Endocortical perimeter (Ec.Pm) (E) Total area (Tt.Ar) (F) Medullary area (Ma.Ar) (G) Cortical TMD (g/cm^3^). Data represented as mean±SEM. Significant differences between timepoints (main effect for time) are denoted with lowercase letters; Significant differences between timepoints within a group (interaction effect) are denoted with Greek letters for placebo group and uppercase letters for LPS group.

In the female mice, there was no significant main effect for LPS treatment, but there was a main effect for time, reflecting an overall increase in Ct.Ar/Tt.Ar (*p* < 0.01; Fig 7A), Ct.Th (*p* < 0.01; Fig 7B), Ps.Pm (*p* < 0.01; 7C), Tt.Ar (*p* < 0.01; 7E), and TMD (*p* < 0.01; Fig 7G), and an overall decrease in Ec.Pm (*p* < 0.01; 7D) and Ma.Ar (*p* < 0.01; 7F). In addition, there was a significant LPS x time interaction for Ps.Pm (*p* < 0.01; Fig 7C), Ec.Pm (*p* < 0.01; Fig 7D), Tt.Ar (*p* < 0.01; Fig 7E), and Ma.Ar (*p* < 0.05; Fig 7F). Post-hoc comparisons revealed that the placebo and LPS groups tracked each other, with the exception of Tt.At which only increased in the LPS group but not in the placebo, and both Ec.Pm and Ma.Ar which only decreased in the placebo group but not the LPS from 8-weeks to 20-weeks of age.

## Discussion

In this study, we investigated the effects of chronic LPS exposure delivered via Alzet osmotic pumps on trabecular and cortical bone structure and BMD in male and female CD-1 mice. We selected a duration of 12-weeks to allow for changes in bone structure and density to establish the time-course effect of LPS longitudinally. Since there was no dose-dependent serum LPS response, the low, mid, and high LPS groups were combined for analysis. Although serum LPS was elevated in the combined LPS group compared to the placebo at the end of the 12-week study in both male and female mice, this was not sufficient to induce either an inflammatory response based on blood smear leukocyte counts or negatively impact trabecular and cortical bone outcomes in either sex. However, based on previous studies [33–36] during this life period from 8 to 20 weeks of age we did observe the expected alterations in bone outcomes with an age-related decline in trabecular bone structure and BMD and increase in cortical bone structure and TMD.

Although there is no set cut-off for the concentration of circulating LPS that alters bone structure or BMD, it remains well recognized as an underlying cause to many diseases including osteoporosis [2–6]. The immune system can be activated by LPS, which elicits a whole-body response inducing increased circulating pro-inflammatory cytokines [12]. Previous studies have demonstrated that chronic LPS exposure induces a pro-inflammatory state that can negatively affect bone metabolism to favour resorption, resulting in compromised bone structure in rodent models [13–18]. Although we were unable to measure circulating pro-inflammatory cytokines in the present study, the elevated serum LPS concentrations (males 0.63 ng/mL; female 0.54 ng/mL) did not reach the threshold to negatively impact bone outcomes.

The lack of LPS-induced alterations on bone outcomes in our study could be explained by the delivery system. Previous research investigating the influence of LPS on bone structure has used slow-release pellets [13–18]. In a study by Droke et al. [13] they first established the LPS dose required for chronic inflammation induced bone and cardiovascular deterioration. Based on blood smears the mid-dose (1.33 μg/day) was established to induce the greatest difference in the ratio of lymphocytes to neutrophils with no further change with the high-dose (13.3 μg/day). Although we based our LPS dosage on this previous work, we found no differences in blood smear leukocytes between any of the groups. Trabecular bone structure at the distal femur metaphysis, but not cortical bone at the femur mid-diaphysis were compromised after 4-weeks of LPS exposure. The negative effects of LPS induced alterations in bone structure may be explained by the upregulation of TNF-α expression in the tibia metaphyseal region that could impair bone turnover [13].

Other studies have reported similar results using subcutaneously implanted LPS slow-release pellets in both 14-week old male C57BL/6J mice at a dose of 0.1 mg/kg body weight/day (approximately 2.5 μg/day estimated for a 25g mouse) for 4 weeks [14] and 6-week old female C57Bl/6J mice at a dose of 1.5 μg/day for 13 weeks [15]. More specifically, 4-weeks of LPS exposure in male C57BL/6J mice resulted in reduced vertebral body BMD, BV/TV, and Tb.N, which led to reduced bone strength evaluated using finite element analysis. Interestingly, unlike the vertebral body, the reduction in BV/TV at the proximal tibia metaphysis did not reach statistical significance, but proximal tibia strength was still reduced. This 4-week exposure to LPS did not alter cortical bone outcomes [14]. In the other study, Cao et al. [15] explained the compromised bone structure at the distal femur and second lumbar vertebra induced by 13-weeks of LPS exposure in female C57BL/6J mice as the result of increased osteoclastogenesis and decreased bone formation. Tartrate-resistant phosphatase, a marker of bone resorption, was elevated in the serum along with an increase in bone marrow osteoclast differentiation. Compounding the elevated bone resorption, bone formation was suggested to be decreased as serum bone-specific alkaline phosphatase concentration was reduced [15].

Altered bone formation and resorption can translate to alterations in trabecular bone structure, as demonstrated in Sprague-Dawley rats, implanted with LPS slow-release pellets. LPS caused an up-regulation of proinflammatory cytokines, IL-1 and TNF-α, resulting in a reduced BV/TV, Tb.N, and an increased Tb.Sp, which translated to impaired biomechanical strength [16]. Similarly, a decrease in circulating osteocalcin and increased tartrate-resistant phosphatase along with a decrease in femur bone mineral content and BMD have been reported in response to 12-week LPS exposure in 12-week old female CD rats [17]. It is important to note that these previous studies only analyzed bone outcomes *ex vivo* using either histomorphometric analysis, dual energy X-ray absorptiometry, or μCT. In the present study, we used *in vivo* analysis to track bone outcomes longitudinally to better understand how bone is altered with LPS exposure and over time. Performing multiple scans allowed us to determine that the placebo and LPS groups did not differ over the course of the 12-week LPS treatment at any of the scanning timepoints–at 8, 12, 16, or 20 weeks of age. However with time, outcomes of both trabecular and cortical bones were altered and the direction of these changes were expected based on previous studies by our lab [33,34] and others [35,36]. The CD-1 mouse strain used for this study may in part explain our findings due to the inherent variability of an outbred strain. Although LPS was demonstrated to negatively alter bone outcomes in the outbred Sprague-Dawley and CD rats [16,17], these results may not be transferable between species and requires more investigation. Previous studies in mice have used in the inbred C57BL/6J strain [13–15] and this may account for some of the differences in our results.

Multiple injections to deliver LPS longitudinally are not preferable since the half-life of LPS is suggested to be around 12 hours [37]. Although no studies have specifically investigated the release rate of LPS from slow-release pellets, previous work has demonstrated that pellets containing 17β-estradiol had an initial supraphysiological release followed by a substantial decline in both 42-day [20] and 90-day interventions [21]. This would suggest that the LPS pellets used in previous studies may provide an initial bolus followed by a substantial decline which may not be representative of delivering a continuous dose of LPS over time, though it is important to note that an inflammatory response was still observed. Whereas, Alzet osmotic pumps have a very consistent release rate [23,24]. Combining the low, mid, and high LPS groups for analysis due to the lack of dose-response is a limitation in the present study. Although the LPS dosages were modelled after previous studies using slow-release pellets [13], the present study highlights that greater variations between dosages may be required when using osmotic pumps for delivery. To better understand administering LPS via osmotic pumps and the half-life of LPS, serum LPS should be measured longitudinally. This was not feasible in the present study due to the amount of blood that would be required. Future studies should compare osmotic pumps versus slow-release pellets to address LPS release rate and establish a representative model of chronic low-grade inflammation. Additionally, circulating LPS and pro-inflammatory cytokines should be analyzed at different time points to ensure there is a consistent inflammatory response. This proves difficult with mice as the amount of serum required using currently available kits can only be obtained upon euthanasia. In conclusion, despite the elevated serum LPS in both the male and female CD-1 mice, a 12-week exposure to LPS delivered via osmotic pumps did not compromise trabecular or cortical bone outcomes.

## Supporting information

S1 Table Serum lipopolysaccharide raw data. (DOCX)

S2 Table Blood smear leukocyte counts raw data. (DOCX)

S3 Table Body weight raw data. (DOCX)

S4 Table Trabecular bone raw data. (DOCX)

S5 Table Cortical bone raw data. (DOCX)
