# Supplementary material for: Trabecular and cortical bone are unaltered in response to chronic lipopolysaccharide exposure via osmotic pumps in male and female CD-1 mice
Source: PLoS One. 2021 Feb 5;16(2):e0243933. doi: 10.1371/journal.pone.0243933 (PMC7864436; doi:10.1371/journal.pone.0243933)
Supplement: S1 Table — (DOCX) [file pone.0243933.s001.docx]

**Serum Lipopolysaccharide**

- All measures in ng/mL

**Males**

|  | **Placebo** | **Low (0.9 μg/d)** | **Mid (3.6 μg/d)** | **High (14.4 μg/d)** |
| --- | --- | --- | --- | --- |
|  | 0.36 | 0.50 | 0.53 | 1.37 |
|  | 0.34 | 1.11 | 0.49 | 0.73 |
|  | 0.54 | 0.46 | 0.58 | 0.36 |
|  | 0.38 | 0.70 | 0.58 | 0.36 |
|  |  |  |  | 0.44 |
| **Average** | **0.40** | **0.69** | **0.54** | **0.65** |
| **St.Dev** | **0.09** | **0.30** | **0.04** | **0.43** |

**Females**

|  | **Placebo** | **Low (0.9 μg/d)** | **Mid (3.6 μg/d)** | **High (14.4 μg/d)** |
| --- | --- | --- | --- | --- |
|  | 0.15 | 0.40 | 0.71 | 0.34 |
|  | 0.30 | 0.55 | 0.59 | 0.42 |
|  | 0.33 | 0.43 | 0.66 | 0.38 |
|  | 0.22 | 1.03 | 0.35 | 0.65 |
|  | 0.16 | 0.56 | 0.42 | 0.63 |
| **Average** | **0.23** | **0.59** | **0.54** | **0.48** |
| **St.Dev** | **0.08** | **0.26** | **0.15** | **0.14** |
