# Supplementary material for: Trabecular and cortical bone are unaltered in response to chronic lipopolysaccharide exposure via osmotic pumps in male and female CD-1 mice
Source: PLoS One. 2021 Feb 5;16(2):e0243933. doi: 10.1371/journal.pone.0243933 (PMC7864436; doi:10.1371/journal.pone.0243933)
Supplement: S2 Table — (DOCX) [file pone.0243933.s002.docx]

**Blood Smear Leukocyte Counts**

- Averaged number of leukocytes per field of view – for a total of 10 fields of view

**Males**

|  | **Placebo** | **LPS** |
| --- | --- | --- |
|  | 1.2 | 0.6 |
|  | 0.2 | 0.3 |
|  | 0.2 | 0.8 |
|  | 2.2 | 1.1 |
|  | 1.4 | 1.1 |
|  | 1.9 | 0.8 |
|  | 1.3 | 0.4 |
|  | 1.9 | 0.7 |
|  | 0.6 | 0.4 |
|  |  | 1.5 |
|  |  | 0.3 |
|  |  | 0.1 |
|  |  | 2.3 |
|  |  | 1.8 |
|  |  | 0.9 |
|  |  | 1.4 |
|  |  | 1.2 |
| **Average** | **1.2** | **0.9** |
| **St.Dev** | **0.7** | **0.6** |

**Females**

|  | **Placebo** | **LPS** |
| --- | --- | --- |
|  | 1.1 | 2 |
|  | 1.5 | 0.6 |
|  | 1.3 | 1.7 |
|  | 1.6 | 1.3 |
|  | 2.2 | 1.9 |
|  | 1.8 | 1.3 |
|  | 1.0 | 1.8 |
|  | 1.1 | 2.0 |
|  | 1.1 | 0.4 |
|  | 2.3 | 0.8 |
|  | 3.3 | 0.9 |
|  |  | 1.4 |
|  |  | 0.6 |
|  |  | 3.5 |
|  |  | 1.3 |
|  |  | 1.5 |
|  |  | 1.7 |
|  |  | 1.1 |
|  |  | 0.3 |
|  |  | 1.0 |
|  |  | 1.4 |
|  |  | 0.6 |
|  |  | 1.2 |
|  |  | 3.4 |
|  |  | 1.2 |
|  |  | 0.4 |
|  |  | 1.3 |
| **Average** | **1.7** | **1.4** |
| **St.Dev** | **0.7** | **0.8** |
