# Supplementary material for: Trabecular and cortical bone are unaltered in response to chronic lipopolysaccharide exposure via osmotic pumps in male and female CD-1 mice
Source: PLoS One. 2021 Feb 5;16(2):e0243933. doi: 10.1371/journal.pone.0243933 (PMC7864436; doi:10.1371/journal.pone.0243933)
Supplement: S3 Table — (DOCX) [file pone.0243933.s003.docx]

**Body Weight**

- All measures in grams
- Each row represents an individual animal and columns represent timepoints

**Males**

|  | **8 weeks** |  |  |  | **12 weeks** |  |  |  | **16 weeks** |  |  |  | **20 weeks** |
| --- | --- | --- | --- | --- | --- | --- | --- | --- | --- | --- | --- | --- | --- |
| **Placebo** | 30.5 | 33.0 | 33.8 | 34.6 | 34.1 | 34.4 | 34.2 | 35.0 | 34.8 | 34.4 | 36.1 | 35.9 | 35.3 |
|  | 29.3 | 32.5 | 33.7 | 32.8 | 32.2 | 33.5 | 32.4 | 31.6 | 33.2 | 33.5 | 33.5 | 31.9 | 33.4 |
|  | 31.2 | 34.9 | 34.5 | 34.8 | 34.7 | 35.9 | 35.6 | 36.4 | 36.1 | 36.8 | 36.7 | 37.4 | 37.0 |
|  | 32.2 | 34.6 | 33.2 | 33.3 | 34.2 | 36.4 | 35.8 | 36.6 | 38.6 | 37.1 | 37.6 | 37.2 | 36.6 |
|  | 35.1 | 37.6 | 35.3 | 37.6 | 41.2 | 41.3 | 39.2 | 40.8 | 39.5 | 40.1 | 40.7 | 39.8 | 40.9 |
|  | 30.8 | 31.9 | 32.8 | 33.5 | 32.7 | 34.4 | 33.6 | 34.2 | 33.5 | 33.2 | 34.2 | 36.5 | 35.8 |
|  | 36.3 | 40.6 | 40.1 | 42.4 | 46.2 | 46.5 | 50.3 | 52.5 | 50.6 | 50.7 | 55.2 | 56.4 | 55.5 |
|  | 34.0 | 37.5 | 38.4 | 38.6 | 39.6 | 39.8 | 39.7 | 40.8 | 39.8 | 40.8 | 42.1 | 43.2 | 42.1 |
|  | 35.7 | 37.1 | 34.9 | 34.4 | 35.6 | 38.2 | 37.3 | 38.6 | 35.6 | 37.8 | 38.9 | 39.0 | 37.8 |
|  | 32.6 | 36.0 | 35.6 | 36.9 | 36.2 | 38.1 | 37.6 | 38.0 | 37.2 | 36.8 | 37.9 | 38.3 | 37.7 |
|  | 30.8 | 34.8 | 34.0 | 35.9 | 35.8 | 37.2 | 36.5 | 35.5 | 35.4 | 35.7 | 38.1 | 39.3 | 38.8 |
|  | 34.3 | 37.7 | 35.3 | 35.3 | 36.1 | 37.1 | 37.3 | 39.1 | 37.7 | 36.7 | 39.2 | 39.8 | 38.9 |

|  | **8 weeks** |  |  |  | **12 weeks** |  |  |  | **16 weeks** |  |  |  | **20 weeks** |
| --- | --- | --- | --- | --- | --- | --- | --- | --- | --- | --- | --- | --- | --- |
| **LPS** | 30.1 | 30.8 | 30.2 | 29.5 | 30.6 | 30.8 | 30.3 | 30.2 | 30.0 | 29.1 | 29.6 | 30.0 | 29.8 |
|  | 31.2 | 32.5 | 32.6 | 33.4 | 34.2 | 34.7 | 34.5 | 33.1 | 33.3 | 34.0 | 33.7 | 35.3 | 34.2 |
|  | 29.4 | 32.9 | 34.9 | 34.4 | 33.2 | 33.4 | 33.2 | 33.4 | 34.1 | 33.0 | 33.8 | 33.8 | 33.3 |
|  | 28.3 | 30.5 | 31.2 | 31.4 | 31.0 | 31.7 | 31.1 | 31.8 | 31.3 | 31.9 | 33.3 | 33.7 | 32.8 |
|  | 36.0 | 38.0 | 37.5 | 38.6 | 39.2 | 39.8 | 41.6 | 41.7 | 41.7 | 41.1 | 43.3 | 43.1 | 44.1 |
|  | 36.0 | 35.7 | 35.7 | 38.5 | 39.2 | 38.9 | 39.4 | 39.3 | 39.9 | 40.3 | 42.1 | 41.9 | 42.5 |
|  | 33.0 | 35.8 | 36.8 | 37.0 | 37.7 | 38.3 | 39.0 | 39.1 | 39.1 | 40.4 | 41.7 | 42.2 | 43.3 |
|  | 29.8 | 33.0 | 32.9 | 34.3 | 34.4 | 36.1 | 36.1 | 36.7 | 37.3 | 36.2 | 37.3 | 38.3 | 38.6 |
|  | 34.6 | 35.5 | 36.6 | 37.8 | 38.0 | 38.5 | 38.5 | 39.1 | 39.4 | 39.8 | 41.3 | 42.8 | 41.1 |
|  | 32.3 | 34.1 | 35.2 | 36.8 | 36.2 | 36.9 | 36.3 | 36.4 | 36.6 | 36.5 | 37.8 | 36.2 | 35.8 |
|  | 30.6 | 32.7 | 33.2 | 33.7 | 33.8 | 34.4 | 34.3 | 34.4 | 34.5 | 34.5 | 35.7 | 36.7 | 34.4 |
|  | 27.0 | 29.1 | 28.8 | 29.3 | 30.3 | 31.2 | 32.0 | 31.9 | 32.5 | 32.4 | 33.0 | 33.1 | 33.7 |
|  | 33.2 | 33.6 | 33.7 | 33.8 | 34.4 | 33.9 | 34.2 | 33.7 | 34.3 | 34.8 | 35.0 | 35.5 | 35.7 |
|  | 28.5 | 30.6 | 30.7 | 30.4 | 31.6 | 31.6 | 31.4 | 31.2 | 31.4 | 32.1 | 33.6 | 32.8 | 33.3 |
|  | 30.7 | 33.5 | 34.3 | 35.9 | 36.6 | 37.9 | 37.2 | 39.1 | 37.7 | 39.6 | 40.1 | 42.1 | 42.5 |
|  | 33.4 | 34.0 | 34.4 | 33.9 | 34.1 | 35.7 | 34.3 | 35.5 | 34.7 | 35.8 | 36.2 | 40.1 | 37.4 |
|  | 33.5 | 36.3 | 37.4 | 37.0 | 38.1 | 38.2 | 37.2 | 38.2 | 38.8 | 38.8 | 39.7 | 38.0 | 38.2 |
|  | 37.9 | 36.9 | 39.0 | 38.6 | 41.4 | 40.5 | 42.1 | 41.4 | 43.5 | 40.1 | 42.7 | 44.1 | 46.4 |
|  | 32.6 | 30.3 | 35.3 | 35.7 | 37.2 | 39.5 | 38.6 | 40.1 | 40.5 | 39.1 | 40.6 | 44.2 | 41.5 |
|  | 29.9 | 30.2 | 31.7 | 32.2 | 33.6 | 33.7 | 33.4 | 33.7 | 34.2 | 34.1 | 35.3 | 36.7 | 35.3 |
|  | 30.2 | 30.8 | 31.5 | 31.5 | 32.4 | 33.6 | 32.3 | 32.7 | 32.8 | 33.0 | 34.7 | 34.8 | 34.3 |
|  | 29.6 | 32.6 | 33.2 | 33.7 | 34.1 | 34.5 | 34.5 | 34.5 | 35.2 | 34.3 | 35.1 | 35.7 | 37.0 |
|  | 29.8 | 31.5 | 33.8 | 32.9 | 34.3 | 34.2 | 33.2 | 34.8 | 34.5 | 34.8 | 36.7 | 35.7 | 36.4 |
|  | 30.5 | 32.2 | 32.8 | 33.2 | 35.4 | 36.0 | 35.0 | 35.5 | 36.0 | 36.8 | 36.2 | 36.9 | 37.6 |
|  | 35.6 | 38.1 | 35.6 | 36.5 | 37.5 | 40.3 | 39.7 | 41.3 | 41.0 | 41.1 | 43.3 | 43.5 | 43.5 |
|  | 31.5 | 33.0 | 32.9 | 34.2 | 34.1 | 36.3 | 36.5 | 36.8 | 35.8 | 33.4 | 36.7 | 36.7 | 36.6 |
|  | 31.6 | 30.6 | 32.8 | 34.3 | 35.3 | 36.2 | 35.8 | 36.1 | 35.4 | 36.5 | 36.6 | 36.8 | 36.8 |
|  | 32.5 | 31.8 | 33.6 | 36.0 | 36.4 | 38.5 | 38.8 | 38.4 | 39.7 | 37.9 | 39.4 | 41.0 | 41.3 |
|  | 35.5 | 34.6 | 36.8 | 38.5 | 39.6 | 39.8 | 40.5 | 40.4 | 40.3 | 40.8 | 41.3 | 41.1 | 40.8 |
|  | 33.4 | 30.8 | 34.5 | 35.8 | 34.5 | 37.8 | 37.8 | 39.2 | 37.9 | 39.2 | 40.6 | 41.7 | 42.8 |

**Females**

|  | **8 weeks** |  |  |  | **12 weeks** |  |  |  | **16 weeks** |  |  |  | **20 weeks** |
| --- | --- | --- | --- | --- | --- | --- | --- | --- | --- | --- | --- | --- | --- |
| **Placebo** | 28.1 | 29.6 | 29.6 | 30.8 | 32.5 | 31.2 | 33.6 | 33.2 | 35.0 | 34.8 | 32.6 | 35.6 | 34.2 |
|  | 28.1 | 30.4 | 28.8 | 29.1 | 30.0 | 30.8 | 30.4 | 33.8 | 32.9 | 31.9 | 34.7 | 34.3 | 34.4 |
|  | 27.2 | 29.1 | 28.9 | 29.5 | 29.7 | 33.4 | 31.4 | 37.1 | 38.6 | 37.1 | 36.4 | 38.1 | 39.6 |
|  | 25.9 | 28.6 | 29.5 | 30.0 | 31.2 | 31.6 | 30.9 | 33.9 | 33.4 | 32.1 | 32.2 | 31.5 | 33.1 |
|  | 28.3 | 28.7 | 30.3 | 34.2 | 32.1 | 31.4 | 33.8 | 33.4 | 35.1 | 33.0 | 34.0 | 34.5 | 36.6 |
|  | 28.2 | 30.1 | 33.0 | 32.7 | 31.9 | 32.4 | 32.4 | 35.1 | 36.1 | 33.5 | 35.2 | 36.5 | 35.6 |
|  | 28.8 | 30.3 | 31.0 | 32.4 | 32.8 | 36.0 | 35.1 | 40.7 | 40.8 | 41.7 | 41.8 | 46.5 | 47.4 |
|  | 27.6 | 29.4 | 29.4 | 29.8 | 30.0 | 29.8 | 31.5 | 33.2 | 32.8 | 33.1 | 33.1 | 33.2 | 34.0 |
|  | 24.8 | 25.7 | 27.4 | 29.5 | 29.4 | 29.8 | 30.1 | 32.8 | 31.3 | 27.2 | 31.0 | 33.3 | 32.9 |
|  | 26.6 | 26.8 | 27.5 | 28.4 | 30.9 | 30.2 | 31.4 | 32.3 | 32.1 | 30.8 | 31.9 | 34.7 | 34.8 |
|  | 24.0 | 26.7 | 27.0 | 27.0 | 29.0 | 28.2 | 27.2 | 29.2 | 28.7 | 30.5 | 29.7 | 28.9 | 30.2 |

|  | **8 weeks** |  |  |  | **12 weeks** |  |  |  | **16 weeks** |  |  |  | **20 weeks** |
| --- | --- | --- | --- | --- | --- | --- | --- | --- | --- | --- | --- | --- | --- |
| **LPS** | 25.2 | 22.7 | 27.9 | 29.1 | 31.7 | 29.8 | 31.0 | 32.7 | 32.9 | 33.0 | 34.1 | 34.2 | 33.5 |
|  | 26.6 | 26.3 | 29.6 | 32.5 | 34.8 | 33.5 | 35.1 | 34.4 | 34.7 | 34.3 | 37.2 | 35.4 | 34.6 |
|  | 25.5 | 26.4 | 29.9 | 29.7 | 31.1 | 29.6 | 33.4 | 33.4 | 31.5 | 32.1 | 31.8 | 33.1 | 32.9 |
|  | 28.6 | 30.7 | 32.5 | 32.5 | 33.5 | 32.7 | 32.7 | 34.8 | 39.5 | 37.1 | 36.0 | 37.1 | 37.0 |
|  | 25.0 | 24.0 | 28.2 | 30.5 | 30.3 | 29.1 | 29.8 | 31.0 | 31.4 | 30.3 | 31.1 | 32.4 | 34.5 |
|  | 29.1 | 28.9 | 29.5 | 21.8 | 34.2 | 33.4 | 34.7 | 34.2 | 36.4 | 34.5 | 35.8 | 34.4 | 37.8 |
|  | 25.8 | 23.4 | 31.6 | 28.9 | 31.7 | 30.2 | 29.7 | 31.7 | 31.6 | 31.1 | 29.9 | 30.8 | 31.9 |
|  | 28.4 | 29.1 | 27.9 | 35.6 | 35.7 | 33.9 | 35.1 | 36.6 | 40.2 | 37.2 | 35.1 | 38.8 | 36.8 |
|  | 26.2 | 27.5 | 27.8 | 28.5 | 33.1 | 32.9 | 34.1 | 35.0 | 34.4 | 37.1 | 37.1 | 42.4 | 39.1 |
|  | 25.3 | 24.4 | 27.4 | 28.8 | 30.3 | 30.3 | 32.1 | 31.1 | 32.6 | 31.0 | 30.8 | 33.6 | 33.2 |
|  | 29.8 | 27.5 | 31.5 | 32.3 | 33.2 | 34.4 | 37.8 | 37.7 | 41.6 | 39.1 | 38.4 | 40.5 | 38.7 |
|  | 30.8 | 28.6 | 31.4 | 33.9 | 35.5 | 35.4 | 37.2 | 40.7 | 45.1 | 41.1 | 42.5 | 45.5 | 46.5 |
|  | 29.4 | 27.2 | 30.8 | 31.8 | 36.3 | 34.4 | 34.2 | 37.2 | 36.1 | 34.2 | 36.7 | 37.1 | 35.8 |
|  | 25.4 | 25.0 | 29.9 | 30.9 | 31.8 | 32.1 | 31.6 | 33.2 | 34.3 | 35.1 | 34.2 | 35.4 | 36.4 |
|  | 28.1 | 25.7 | 30.5 | 31.0 | 30.7 | 31.4 | 31.7 | 34.4 | 33.9 | 33.1 | 34.1 | 33.4 | 32.5 |
|  | 26.1 | 25.5 | 27.9 | 29.1 | 30.2 | 29.6 | 30.1 | 32.4 | 31.7 | 33.2 | 32.3 | 33.8 | 31.6 |
|  | 26.7 | 25.9 | 29.7 | 31.9 | 30.3 | 29.8 | 31.2 | 34.3 | 31.9 | 33.8 | 31.9 | 32.9 | 34.7 |
|  | 28.5 | 25.8 | 28.2 | 29.5 | 31.7 | 32.8 | 34.3 | 36.8 | 38.4 | 38.7 | 39.5 | 40.4 | 45.1 |
|  | 29.2 | 25.9 | 30.3 | 32.8 | 33.4 | 34.1 | 35.2 | 38.2 | 34.5 | 37.9 | 35.0 | 40.2 | 38.6 |
|  | 23.7 | 21.4 | 25.6 | 26.7 | 27.8 | 28.5 | 30.7 | 29.4 | 29.4 | 30.6 | 33.4 | 31.5 | 32.6 |
|  | 27.6 | 24.8 | 28.3 | 30.7 | 30.8 | 30.6 | 31.2 | 31.9 | 34.6 | 32.3 | 32.2 | 36.4 | 33.7 |
|  | 27.5 | 24.8 | 28.6 | 29.4 | 29.8 | 30.3 | 29.9 | 31.6 | 31.5 | 31.9 | 31.3 | 32.1 | 32.8 |
|  | 26.2 | 25.1 | 28.1 | 28.9 | 30.3 | 30.7 | 30.6 | 32.0 | 31.8 | 32.8 | 31.4 | 33.6 | 33.4 |
|  | 27.0 | 26.1 | 28.2 | 31.4 | 30.9 | 31.3 | 33.0 | 33.2 | 35.1 | 33.2 | 33.4 | 37.4 | 34.5 |
|  | 27.4 | 25.3 | 28.1 | 30.1 | 31.2 | 30.2 | 30.9 | 31.6 | 32.7 | 35.3 | 32.6 | 34.2 | 32.9 |
|  | 26.6 | 26.8 | 30.2 | 29.5 | 32.9 | 31.9 | 33.8 | 32.2 | 33.4 | 33.1 | 33.2 | 34.6 | 37.2 |
|  | 26.9 | 26.0 | 27.5 | 29.2 | 33.1 | 32.0 | 34.2 | 35.0 | 39.2 | 38.2 | 38.1 | 42.1 | 40.4 |
