# Supplementary material for: Trabecular and cortical bone are unaltered in response to chronic lipopolysaccharide exposure via osmotic pumps in male and female CD-1 mice
Source: PLoS One. 2021 Feb 5;16(2):e0243933. doi: 10.1371/journal.pone.0243933 (PMC7864436; doi:10.1371/journal.pone.0243933)
Supplement: S4 Table — (DOCX) [file pone.0243933.s004.docx]

**Trabecular Bone**

- Each row represents an individual animal and columns represent timepoints

**Males**

**BV/TV (%)**

|  | **8 weeks** | **12 weeks** | **16 weeks** | **20 weeks** |  | **8 weeks** | **12 weeks** | **16 weeks** | **20 weeks** |
| --- | --- | --- | --- | --- | --- | --- | --- | --- | --- |
| **Placebo** | 19.81 | 5.59 | 5.46 | 5.57 | **LPS** | 23.47 | 9.94 | 6.89 | 6.01 |
|  | 16.46 | 8.88 | 8.13 | 7.16 |  | 33.76 | 12.76 | 9.71 | 9.11 |
|  | 20.02 | 8.32 | 6.51 | 4.47 |  | 14.96 | 5.63 | 4.22 | 3.26 |
|  | - | 8.28 | 6.65 | 3.78 |  | 29.23 | 15.93 | 14.91 | 10.19 |
|  | 20.96 | 8.61 | 5.57 | 5.20 |  | 36.96 | 20.32 | 16.87 | 17.73 |
|  | 9.85 | 4.31 | 3.80 | 4.00 |  | 30.51 | 16.58 | 12.05 | 11.90 |
|  | 16.31 | 8.62 | 5.27 | 6.06 |  | 30.46 | 14.77 | 10.43 | 9.31 |
|  | 27.68 | 9.74 | 7.31 | 7.76 |  | 9.52 | 4.60 | 2.93 | 4.95 |
|  | 20.61 | 5.18 | 5.21 | 5.76 |  | 15.71 | 5.56 | 4.39 | 6.22 |
|  | 25.93 | 14.42 | 12.43 | 14.09 |  | 17.49 | 7.55 | 4.70 | 5.15 |
|  | 13.12 | 9.99 | 8.42 | 9.87 |  | 25.52 | 16.60 | 12.98 | 12.27 |
|  | 21.34 | 9.65 | 5.11 | 7.13 |  | 14.62 | 4.51 | 1.98 | 1.73 |
| **Avg** | **19.28** | **8.47** | **6.66** | **6.74** |  | 34.53 | 15.73 | 14.12 | 13.48 |
| **St.Dev** | **5.18** | **2.65** | **2.26** | **2.89** |  | 32.53 | 12.41 | 9.45 | 7.82 |
|  | | | | |  | 19.13 | 7.61 | 3.40 | 2.58 |
|  |  |  |  |  |  | 13.92 | 7.64 | 6.08 | 5.90 |
|  |  |  |  |  |  | 22.73 | 10.88 | 8.42 | 8.98 |
|  |  |  |  |  |  | 21.68 | 8.87 | 8.22 | 10.50 |
|  |  |  |  |  |  | 25.19 | 17.47 | 26.21 | 18.94 |
|  |  |  |  |  |  | 8.56 | 4.70 | 2.53 | 2.83 |
|  |  |  |  |  |  | 17.48 | 5.27 | 3.39 | 2.31 |
|  |  |  |  |  |  | 29.57 | 11.03 | 7.33 | 4.27 |
|  |  |  |  |  |  | 15.16 | 5.44 | 2.75 | 2.45 |
|  |  |  |  |  |  | 11.51 | 2.64 | 1.08 | 1.10 |
|  |  |  |  |  |  | 25.24 | 7.20 | 3.58 | 3.04 |
|  |  |  |  |  |  | 33.59 | 6.44 | 3.91 | 4.48 |
|  |  |  |  |  |  | 33.66 | 14.50 | 12.33 | 11.89 |
|  |  |  |  |  |  | 15.34 | 6.11 | 3.62 | 5.98 |
|  |  |  |  |  |  | 23.63 | 11.75 | 7.80 | 9.15 |
|  |  |  |  |  |  | 20.59 | 10.51 | 7.73 | 8.07 |
|  |  |  |  |  | **Avg** | **22.87** | **10.03** | **7.80** | **7.39** |
|  |  |  |  |  | **St.Dev** | **8.24** | **4.76** | **5.49** | **4.59** |

**Tb.N (mm^-1^)**

|  | **8 weeks** | **12 weeks** | **16 weeks** | **20 weeks** |  | **8 weeks** | **12 weeks** | **16 weeks** | **20 weeks** |
| --- | --- | --- | --- | --- | --- | --- | --- | --- | --- |
| **Placebo** | 2.59 | 0.81 | 0.77 | 0.75 | **LPS** | 2.56 | 1.20 | 0.84 | 0.64 |
|  | 2.08 | 1.05 | 0.96 | 0.89 |  | 3.24 | 1.44 | 1.07 | 0.99 |
|  | 2.38 | 1.04 | 0.82 | 0.59 |  | 1.97 | 0.69 | 0.56 | 0.45 |
|  | - | 1.22 | 1.00 | 0.53 |  | 3.20 | 1.68 | 1.64 | 1.14 |
|  | 2.62 | 1.14 | 0.66 | 0.58 |  | 4.23 | 2.71 | 2.24 | 2.13 |
|  | 1.33 | 0.59 | 0.52 | 0.48 |  | 3.47 | 1.95 | 1.44 | 1.32 |
|  | 2.38 | 1.07 | 0.71 | 0.68 |  | 3.31 | 1.80 | 1.28 | 1.13 |
|  | 3.25 | 1.32 | 0.94 | 0.94 |  | 1.38 | 0.63 | 0.40 | 0.58 |
|  | 2.79 | 0.69 | 0.59 | 0.55 |  | 2.08 | 0.69 | 0.51 | 0.69 |
|  | 3.35 | 1.96 | 1.67 | 1.73 |  | 2.33 | 1.01 | 0.58 | 0.59 |
|  | 2.01 | 1.45 | 1.06 | 1.12 |  | 3.11 | 1.84 | 1.55 | 1.38 |
|  | 2.94 | 1.51 | 0.85 | 1.00 |  | 1.91 | 0.64 | 0.28 | 0.29 |
| **Avg** | **2.52** | **1.15** | **0.88** | **0.82** |  | 3.10 | 1.74 | 1.65 | 1.51 |
| **St.Dev** | **0.58** | **0.38** | **0.30** | **0.35** |  | 3.63 | 1.57 | 1.29 | 1.06 |
|  | | | | |  | 2.44 | 0.97 | 0.48 | 0.35 |
|  |  |  |  |  |  | 1.90 | 0.98 | 0.74 | 0.70 |
|  |  |  |  |  |  | 2.92 | 1.47 | 1.21 | 1.18 |
|  |  |  |  |  |  | 2.77 | 1.19 | 1.15 | 1.22 |
|  |  |  |  |  |  | 3.28 | 2.26 | 2.66 | 2.17 |
|  |  |  |  |  |  | 1.32 | 0.67 | 0.37 | 0.33 |
|  |  |  |  |  |  | 2.14 | 0.56 | 0.43 | 0.36 |
|  |  |  |  |  |  | 3.30 | 1.35 | 0.94 | 0.60 |
|  |  |  |  |  |  | 2.05 | 0.62 | 0.34 | 0.30 |
|  |  |  |  |  |  | 1.53 | 0.34 | 0.15 | 0.16 |
|  |  |  |  |  |  | 2.99 | 0.98 | 0.54 | 0.41 |
|  |  |  |  |  |  | 3.78 | 0.93 | 0.48 | 0.48 |
|  |  |  |  |  |  | 3.80 | 1.77 | 1.56 | 1.40 |
|  |  |  |  |  |  | 2.25 | 0.90 | 0.52 | 0.67 |
|  |  |  |  |  |  | 3.20 | 1.40 | 0.95 | 0.98 |
|  |  |  |  |  |  | 2.83 | 1.32 | 1.05 | 0.93 |
|  |  |  |  |  | **Avg** | **2.74** | **1.24** | **0.96** | **0.87** |
|  |  |  |  |  | **St.Dev** | **0.76** | **0.56** | **0.60** | **0.52** |

**Tb.Th (mm)**

|  | **8 weeks** | **12 weeks** | **16 weeks** | **20 weeks** |  | **8 weeks** | **12 weeks** | **16 weeks** | **20 weeks** |
| --- | --- | --- | --- | --- | --- | --- | --- | --- | --- |
| **Placebo** | 0.076 | 0.069 | 0.071 | 0.075 | **LPS** | 0.092 | 0.083 | 0.082 | 0.094 |
|  | 0.079 | 0.085 | 0.085 | 0.081 |  | 0.104 | 0.089 | 0.091 | 0.092 |
|  | 0.084 | 0.080 | 0.079 | 0.076 |  | 0.076 | 0.082 | 0.076 | 0.072 |
|  | - | 0.068 | 0.066 | 0.071 |  | 0.091 | 0.095 | 0.091 | 0.090 |
|  | 0.080 | 0.075 | 0.085 | 0.089 |  | 0.087 | 0.075 | 0.075 | 0.083 |
|  | 0.074 | 0.073 | 0.074 | 0.083 |  | 0.088 | 0.085 | 0.084 | 0.090 |
|  | 0.069 | 0.080 | 0.074 | 0.089 |  | 0.092 | 0.082 | 0.081 | 0.082 |
|  | 0.085 | 0.074 | 0.078 | 0.083 |  | 0.069 | 0.073 | 0.074 | 0.085 |
|  | 0.074 | 0.076 | 0.088 | 0.104 |  | 0.075 | 0.080 | 0.086 | 0.090 |
|  | 0.077 | 0.074 | 0.074 | 0.081 |  | 0.075 | 0.074 | 0.081 | 0.087 |
|  | 0.065 | 0.069 | 0.080 | 0.088 |  | 0.082 | 0.090 | 0.084 | 0.089 |
|  | 0.073 | 0.064 | 0.060 | 0.071 |  | 0.076 | 0.070 | 0.072 | 0.061 |
| **Avg** | **0.076** | **0.074** | **0.076** | **0.083** |  | 0.111 | 0.090 | 0.086 | 0.089 |
| **St.Dev** | **0.006** | **0.006** | **0.008** | **0.009** |  | 0.090 | 0.079 | 0.073 | 0.074 |
|  | | | | |  | 0.078 | 0.079 | 0.070 | 0.073 |
|  |  |  |  |  |  | 0.073 | 0.078 | 0.082 | 0.085 |
|  |  |  |  |  |  | 0.078 | 0.074 | 0.069 | 0.076 |
|  |  |  |  |  |  | 0.078 | 0.075 | 0.071 | 0.086 |
|  |  |  |  |  |  | 0.077 | 0.077 | 0.098 | 0.087 |
|  |  |  |  |  |  | 0.065 | 0.071 | 0.068 | 0.085 |
|  |  |  |  |  |  | 0.082 | 0.094 | 0.078 | 0.065 |
|  |  |  |  |  |  | 0.090 | 0.082 | 0.078 | 0.072 |
|  |  |  |  |  |  | 0.074 | 0.087 | 0.081 | 0.083 |
|  |  |  |  |  |  | 0.075 | 0.077 | 0.074 | 0.070 |
|  |  |  |  |  |  | 0.084 | 0.074 | 0.067 | 0.074 |
|  |  |  |  |  |  | 0.089 | 0.069 | 0.081 | 0.094 |
|  |  |  |  |  |  | 0.089 | 0.082 | 0.079 | 0.085 |
|  |  |  |  |  |  | 0.068 | 0.068 | 0.070 | 0.089 |
|  |  |  |  |  |  | 0.074 | 0.084 | 0.082 | 0.093 |
|  |  |  |  |  |  | 0.073 | 0.080 | 0.074 | 0.087 |
|  |  |  |  |  | **Avg** | **0.082** | **0.080** | **0.079** | **0.083** |
|  |  |  |  |  | **St.Dev** | **0.010** | **0.007** | **0.008** | **0.009** |

**Tb.Sp (mm)**

|  | **8 weeks** | **12 weeks** | **16 weeks** | **20 weeks** |  | **8 weeks** | **12 weeks** | **16 weeks** | **20 weeks** |
| --- | --- | --- | --- | --- | --- | --- | --- | --- | --- |
| **Placebo** | 0.220 | 0.446 | 0.489 | 0.503 | **LPS** | 0.244 | 0.433 | 0.471 | 0.521 |
|  | 0.273 | 0.376 | 0.437 | 0.474 |  | 0.179 | 0.406 | 0.450 | 0.455 |
|  | 0.268 | 0.440 | 0.474 | 0.534 |  | 0.278 | 0.521 | 0.536 | 0.542 |
|  | - | 0.326 | 0.385 | 0.512 |  | 0.196 | 0.421 | 0.409 | 0.459 |
|  | 0.207 | 0.411 | 0.499 | 0.529 |  | 0.140 | 0.188 | 0.221 | 0.239 |
|  | 0.339 | 0.495 | 0.536 | 0.535 |  | 0.176 | 0.280 | 0.352 | 0.397 |
|  | 0.208 | 0.452 | 0.478 | 0.527 |  | 0.196 | 0.340 | 0.418 | 0.460 |
|  | 0.178 | 0.321 | 0.355 | 0.364 |  | 0.286 | 0.501 | 0.531 | 0.538 |
|  | 0.198 | 0.497 | 0.543 | 0.545 |  | 0.235 | 0.500 | 0.537 | 0.548 |
|  | 0.185 | 0.269 | 0.315 | 0.300 |  | 0.221 | 0.448 | 0.543 | 0.552 |
|  | 0.225 | 0.319 | 0.456 | 0.452 |  | 0.199 | 0.361 | 0.393 | 0.409 |
|  | 0.189 | 0.286 | 0.396 | 0.404 |  | 0.318 | 0.556 | 0.571 | 0.581 |
| **Avg** | **0.226** | **0.387** | **0.447** | **0.473** |  | 0.209 | 0.378 | 0.398 | 0.420 |
| **St.Dev** | **0.049** | **0.081** | **0.071** | **0.079** |  | 0.178 | 0.294 | 0.343 | 0.393 |
|  | | | | |  | 0.237 | 0.423 | 0.491 | 0.552 |
|  |  |  |  |  |  | 0.258 | 0.443 | 0.502 | 0.503 |
|  |  |  |  |  |  | 0.181 | 0.282 | 0.287 | 0.314 |
|  |  |  |  |  |  | 0.180 | 0.442 | 0.456 | 0.452 |
|  |  |  |  |  |  | 0.180 | 0.242 | 0.259 | 0.257 |
|  |  |  |  |  |  | 0.374 | 0.518 | 0.571 | 0.570 |
|  |  |  |  |  |  | 0.243 | 0.539 | 0.559 | 0.549 |
|  |  |  |  |  |  | 0.187 | 0.435 | 0.486 | 0.518 |
|  |  |  |  |  |  | 0.296 | 0.523 | 0.554 | 0.552 |
|  |  |  |  |  |  | 0.372 | 0.559 | 0.576 | 0.574 |
|  |  |  |  |  |  | 0.182 | 0.447 | 0.551 | 0.548 |
|  |  |  |  |  |  | 0.164 | 0.374 | 0.548 | 0.543 |
|  |  |  |  |  |  | 0.160 | 0.281 | 0.329 | 0.334 |
|  |  |  |  |  |  | 0.214 | 0.446 | 0.505 | 0.518 |
|  |  |  |  |  |  | 0.177 | 0.398 | 0.465 | 0.498 |
|  |  |  |  |  |  | 0.212 | 0.391 | 0.438 | 0.474 |
|  |  |  |  |  | **Avg** | **0.222** | **0.412** | **0.458** | **0.476** |
|  |  |  |  |  | **St.Dev** | **0.059** | **0.097** | **0.099** | **0.094** |

**DA (no unit)**

|  | **8 weeks** | **12 weeks** | **16 weeks** | **20 weeks** |  | **8 weeks** | **12 weeks** | **16 weeks** | **20 weeks** |
| --- | --- | --- | --- | --- | --- | --- | --- | --- | --- |
| **Placebo** | 1.95 | 1.96 | 1.97 | 2.03 | **LPS** | 2.03 | 1.90 | 1.77 | 1.80 |
|  | 2.14 | 1.96 | 2.05 | 2.13 |  | 1.84 | 2.33 | 2.01 | 2.09 |
|  | 2.08 | 2.03 | 2.37 | 2.78 |  | 2.64 | 2.59 | 2.84 | 2.59 |
|  | - | 2.00 | 1.99 | 1.88 |  | 2.10 | 1.72 | 1.94 | 1.89 |
|  | 2.01 | 1.85 | 1.62 | 1.63 |  | 2.03 | 1.92 | 1.77 | 1.67 |
|  | 2.28 | 2.05 | 2.01 | 1.67 |  | 1.86 | 1.78 | 1.91 | 1.67 |
|  | 2.04 | 1.92 | 2.09 | 2.03 |  | 2.01 | 2.11 | 2.19 | 2.14 |
|  | 1.79 | 1.77 | 1.77 | 1.62 |  | 2.44 | 2.65 | 2.56 | 1.95 |
|  | 1.93 | 1.98 | 1.72 | 1.67 |  | 2.50 | 2.32 | 2.11 | 1.57 |
|  | 1.97 | 1.85 | 1.83 | 1.81 |  | 2.01 | 1.79 | 1.69 | 1.68 |
|  | 2.13 | 1.92 | 1.87 | 2.00 |  | 1.87 | 1.77 | 1.83 | 1.78 |
|  | 2.27 | 2.39 | 2.43 | 2.39 |  | 2.10 | 2.08 | 2.35 | 2.13 |
| **Avg** | **2.05** | **1.97** | **1.98** | **1.97** |  | 1.87 | 2.02 | 1.87 | 1.83 |
| **St.Dev** | **0.15** | **0.16** | **0.24** | **0.35** |  | 1.80 | 1.75 | 1.74 | 1.84 |
|  | | | | |  | 2.20 | 2.05 | 1.86 | 1.89 |
|  |  |  |  |  |  | 2.06 | 2.09 | 1.77 | 1.66 |
|  |  |  |  |  |  | 2.35 | 2.33 | 2.20 | 1.84 |
|  |  |  |  |  |  | 2.16 | 2.07 | 2.03 | 2.18 |
|  |  |  |  |  |  | 1.99 | 2.05 | 1.65 | 1.81 |
|  |  |  |  |  |  | 2.40 | 2.21 | 1.92 | 1.60 |
|  |  |  |  |  |  | 2.09 | 2.34 | 2.34 | 1.79 |
|  |  |  |  |  |  | 2.07 | 2.17 | 1.95 | 1.94 |
|  |  |  |  |  |  | 2.08 | 2.00 | 1.85 | 1.74 |
|  |  |  |  |  |  | 2.41 | 2.59 | 2.42 | 2.41 |
|  |  |  |  |  |  | 1.95 | 1.85 | 1.85 | 2.13 |
|  |  |  |  |  |  | 1.82 | 1.69 | 2.00 | 1.65 |
|  |  |  |  |  |  | 1.69 | 1.81 | 1.73 | 1.64 |
|  |  |  |  |  |  | 2.10 | 2.18 | 2.08 | 1.75 |
|  |  |  |  |  |  | 2.03 | 1.84 | 1.85 | 1.87 |
|  |  |  |  |  |  | 1.89 | 1.87 | 1.62 | 1.44 |
|  |  |  |  |  | **Avg** | **2.08** | **2.06** | **1.99** | **1.87** |
|  |  |  |  |  | **St.Dev** | **0.23** | **0.27** | **0.28** | **0.25** |

**SMI**

|  | **8 weeks** | **12 weeks** | **16 weeks** | **20 weeks** |  | **8 weeks** | **12 weeks** | **16 weeks** | **20 weeks** |
| --- | --- | --- | --- | --- | --- | --- | --- | --- | --- |
| **Placebo** | 2.21 | 2.79 | 2.71 | 2.60 | **LPS** | 2.11 | 2.46 | 2.79 | 2.98 |
|  | 2.26 | 2.87 | 2.63 | 2.47 |  | 1.73 | 2.41 | 2.48 | 2.51 |
|  | 2.13 | 2.71 | 2.82 | 2.67 |  | 2.40 | 2.72 | 2.51 | 2.66 |
|  | - | 2.60 | 2.67 | 2.84 |  | 1.92 | 2.08 | 2.29 | 2.37 |
|  | 2.28 | 2.52 | 2.85 | 2.65 |  | 1.49 | 2.35 | 2.45 | 2.43 |
|  | 2.56 | 2.77 | 2.72 | 2.85 |  | 1.94 | 2.37 | 2.58 | 2.51 |
|  | 2.46 | 2.58 | 2.82 | 2.80 |  | 1.80 | 2.44 | 2.56 | 2.45 |
|  | 2.04 | 2.63 | 2.78 | 2.85 |  | 2.64 | 2.70 | 2.82 | 2.50 |
|  | 2.24 | 2.65 | 2.63 | 2.68 |  | 2.40 | 2.88 | 2.87 | 2.66 |
|  | 1.85 | 2.41 | 2.50 | 2.48 |  | 2.36 | 2.60 | 2.89 | 2.76 |
|  | 2.47 | 2.55 | 2.50 | 2.45 |  | 2.02 | 2.38 | 2.35 | 2.48 |
|  | 2.18 | 2.53 | 2.66 | 2.58 |  | 2.34 | 2.37 | 2.62 | 2.27 |
| **Avg** | **2.24** | **2.63** | **2.69** | **2.66** |  | 1.78 | 2.38 | 2.30 | 2.32 |
| **St.Dev** | **0.20** | **0.13** | **0.12** | **0.15** |  | 1.69 | 2.56 | 2.52 | 2.59 |
|  | | | | |  | 2.16 | 2.68 | 2.93 | 2.85 |
|  |  |  |  |  |  | 2.50 | 2.53 | 2.68 | 2.57 |
|  |  |  |  |  |  | 2.28 | 2.70 | 2.84 | 2.86 |
|  |  |  |  |  |  | 2.38 | 2.55 | 2.58 | 2.51 |
|  |  |  |  |  |  | 2.09 | 2.38 | 2.06 | 2.53 |
|  |  |  |  |  |  | 2.56 | 2.52 | 2.69 | 2.67 |
|  |  |  |  |  |  | 2.42 | 2.80 | 2.76 | 2.78 |
|  |  |  |  |  |  | 1.86 | 2.36 | 2.56 | 2.54 |
|  |  |  |  |  |  | 2.44 | 2.75 | 2.86 | 2.86 |
|  |  |  |  |  |  | 2.26 | 2.77 | 3.00 | 2.78 |
|  |  |  |  |  |  | 2.22 | 2.54 | 2.47 | 2.89 |
|  |  |  |  |  |  | 1.80 | 2.78 | 2.91 | 2.70 |
|  |  |  |  |  |  | 1.80 | 2.50 | 2.34 | 2.49 |
|  |  |  |  |  |  | 2.44 | 2.61 | 2.77 | 2.70 |
|  |  |  |  |  |  | 2.10 | 2.38 | 2.54 | 2.53 |
|  |  |  |  |  |  | 2.08 | 2.43 | 2.37 | 2.55 |
|  |  |  |  |  | **Avg** | **2.13** | **2.53** | **2.61** | **2.61** |
|  |  |  |  |  | **St.Dev** | **0.30** | **0.18** | **0.23** | **0.18** |

**vBMD (g/cm^3^)**

|  | **8 weeks** | **12 weeks** | **16 weeks** | **20 weeks** |  | **8 weeks** | **12 weeks** | **16 weeks** | **20 weeks** |
| --- | --- | --- | --- | --- | --- | --- | --- | --- | --- |
| **Placebo** | 0.271 | 0.173 | 0.162 | 0.156 | **LPS** | 0.280 | 0.191 | 0.170 | 0.161 |
|  | 0.242 | 0.180 | 0.167 | 0.164 |  | 0.329 | 0.208 | 0.187 | 0.182 |
|  | 0.261 | 0.184 | 0.172 | 0.155 |  | 0.243 | 0.152 | 0.143 | 0.139 |
|  | - | 0.180 | 0.157 | 0.149 |  | 0.311 | 0.222 | 0.219 | 0.190 |
|  | 0.255 | 0.170 | 0.144 | 0.161 |  | 0.330 | 0.261 | 0.241 | 0.245 |
|  | 0.192 | 0.135 | 0.128 | 0.151 |  | 0.307 | 0.225 | 0.197 | 0.207 |
|  | 0.245 | 0.170 | 0.140 | 0.156 |  | 0.302 | 0.211 | 0.185 | 0.190 |
|  | 0.295 | 0.186 | 0.165 | 0.180 |  | 0.187 | 0.129 | 0.116 | 0.145 |
|  | 0.260 | 0.136 | 0.135 | 0.161 |  | 0.238 | 0.145 | 0.134 | 0.161 |
|  | 0.284 | 0.208 | 0.196 | 0.219 |  | 0.237 | 0.157 | 0.128 | 0.151 |
|  | 0.220 | 0.184 | 0.162 | 0.187 |  | 0.294 | 0.233 | 0.211 | 0.203 |
|  | 0.264 | 0.193 | 0.148 | 0.167 |  | 0.226 | 0.143 | 0.123 | 0.125 |
| **Avg** | 0.254 | 0.175 | 0.156 | 0.167 |  | 0.330 | 0.229 | 0.212 | 0.207 |
| **St.Dev** | 0.029 | 0.021 | 0.019 | 0.020 |  | 0.321 | 0.208 | 0.183 | 0.169 |
|  | | | | |  | 0.240 | 0.162 | 0.120 | 0.132 |
|  |  |  |  |  |  | 0.217 | 0.160 | 0.144 | 0.164 |
|  |  |  |  |  |  | 0.268 | 0.200 | 0.172 | 0.192 |
|  |  |  |  |  |  | 0.270 | 0.173 | 0.169 | 0.194 |
|  |  |  |  |  |  | 0.283 | 0.239 | 0.235 | 0.257 |
|  |  |  |  |  |  | 0.184 | 0.130 | 0.113 | 0.133 |
|  |  |  |  |  |  | 0.256 | 0.161 | 0.152 | 0.141 |
|  |  |  |  |  |  | 0.309 | 0.191 | 0.171 | 0.145 |
|  |  |  |  |  |  | 0.245 | 0.153 | 0.135 | 0.134 |
|  |  |  |  |  |  | 0.214 | 0.133 | 0.121 | 0.123 |
|  |  |  |  |  |  | 0.285 | 0.165 | 0.131 | 0.142 |
|  |  |  |  |  |  | 0.320 | 0.164 | 0.137 | 0.156 |
|  |  |  |  |  |  | 0.324 | 0.222 | 0.206 | 0.213 |
|  |  |  |  |  |  | 0.233 | 0.155 | 0.133 | 0.164 |
|  |  |  |  |  |  | 0.274 | 0.185 | 0.163 | 0.184 |
|  |  |  |  |  |  | 0.262 | 0.180 | 0.164 | 0.177 |
|  |  |  |  |  | **Avg** | **0.271** | **0.183** | **0.164** | **0.171** |
|  |  |  |  |  | **St.Dev** | **0.043** | **0.036** | **0.037** | **0.034** |

**Females**

**BV/TV (%)**

|  | **8 weeks** | **12 weeks** | **16 weeks** | **20 weeks** |  | **8 weeks** | **12 weeks** | **16 weeks** | **20 weeks** |
| --- | --- | --- | --- | --- | --- | --- | --- | --- | --- |
| **Placebo** | 21.22 | 15.12 | 8.86 | 5.87 | **LPS** | 15.64 | 3.63 | 4.21 | 2.97 |
|  | 24.42 | 12.50 | 8.20 | 4.96 |  | 16.05 | 6.54 | 6.29 | 5.23 |
|  | 25.69 | 14.35 | 11.54 | 7.43 |  | 14.39 | 4.57 | 5.57 | 3.25 |
|  | 22.06 | 11.82 | 6.52 | 2.70 |  | 20.26 | 12.52 | 13.62 | 11.63 |
|  | 26.40 | 10.19 | 4.19 | 1.93 |  | 19.75 | 8.53 | 7.05 | 5.55 |
|  | 16.63 | 10.54 | 10.21 | 5.54 |  | 38.27 | 19.05 | 17.95 | 11.86 |
|  | 17.21 | 12.11 | 8.44 | 6.11 |  | 41.20 | 14.36 | 10.97 | 6.41 |
|  | 19.06 | 13.10 | 10.69 | 8.02 |  | 19.76 | 10.86 | 8.84 | 5.80 |
|  | 18.99 | 15.02 | 11.11 | 6.66 |  | 21.12 | 12.55 | 10.86 | 7.78 |
|  | 18.30 | 12.29 | 6.31 | 3.38 |  | 23.03 | 7.30 | 6.88 | 4.74 |
|  | 15.43 | 8.22 | 4.31 | 2.87 |  | 24.86 | 13.40 | 12.00 | 8.11 |
| **Avg** | **20.49** | **12.30** | **8.21** | **5.04** |  | 20.12 | 9.66 | 6.04 | 4.26 |
| **St.Dev** | **3.75** | **2.12** | **2.61** | **2.05** |  | 24.77 | 14.31 | 9.45 | 5.67 |
|  | | | | |  | 17.38 | 7.35 | 5.98 | 3.20 |
|  |  |  |  |  |  | 26.42 | 14.64 | 8.91 | 5.31 |
|  |  |  |  |  |  | 18.35 | 6.09 | 3.10 | 2.65 |
|  |  |  |  |  |  | 31.76 | 14.88 | 9.55 | 6.16 |
|  |  |  |  |  |  | 22.63 | 10.03 | 9.71 | 4.50 |
|  |  |  |  |  |  | 14.84 | 8.01 | 7.33 | 3.41 |
|  |  |  |  |  |  | 21.85 | 13.44 | 11.12 | 7.97 |
|  |  |  |  |  |  | 22.83 | 11.20 | 10.48 | 6.53 |
|  |  |  |  |  |  | 26.63 | 15.73 | 16.20 | 11.43 |
|  |  |  |  |  |  | 32.58 | 9.78 | 7.72 | 5.07 |
|  |  |  |  |  |  | 21.67 | 6.80 | 7.25 | 3.19 |
|  |  |  |  |  |  | 21.33 | 12.54 | 10.95 | 7.36 |
|  |  |  |  |  |  | 14.24 | 6.94 | 5.02 | 4.68 |
|  |  |  |  |  |  | 12.68 | 4.86 | 5.29 | 5.30 |
|  |  |  |  |  | **Avg** | **22.38** | **10.35** | **8.83** | **5.93** |
|  |  |  |  |  | **St.Dev** | **7.02** | **3.94** | **3.51** | **2.56** |

**Tb.N (mm^-1^)**

|  | **8 weeks** | **12 weeks** | **16 weeks** | **20 weeks** |  | **8 weeks** | **12 weeks** | **16 weeks** | **20 weeks** |
| --- | --- | --- | --- | --- | --- | --- | --- | --- | --- |
| **Placebo** | 2.05 | 1.45 | 0.85 | 0.56 | **LPS** | 1.86 | 0.43 | 0.44 | 0.34 |
|  | 2.55 | 1.27 | 0.81 | 0.48 |  | 1.91 | 0.74 | 0.69 | 0.55 |
|  | 2.69 | 1.53 | 1.18 | 0.81 |  | 1.71 | 0.58 | 0.61 | 0.38 |
|  | 2.63 | 1.35 | 0.91 | 0.47 |  | 2.17 | 1.25 | 1.27 | 1.09 |
|  | 2.64 | 1.14 | 0.52 | 0.23 |  | 2.31 | 1.02 | 0.79 | 0.59 |
|  | 1.84 | 1.02 | 0.95 | 0.55 |  | 3.30 | 1.74 | 1.61 | 1.08 |
|  | 2.06 | 1.31 | 0.85 | 0.65 |  | 4.30 | 1.54 | 1.20 | 0.73 |
|  | 2.04 | 1.36 | 1.07 | 0.80 |  | 2.13 | 1.20 | 0.94 | 0.59 |
|  | 2.20 | 1.60 | 1.18 | 0.78 |  | 2.58 | 1.39 | 1.11 | 0.75 |
|  | 2.10 | 1.39 | 0.78 | 0.45 |  | 2.48 | 0.84 | 0.74 | 0.53 |
|  | 1.81 | 0.99 | 0.50 | 0.34 |  | 2.69 | 1.54 | 1.34 | 0.97 |
| **Avg** | **2.24** | **1.31** | **0.87** | **0.56** |  | 2.22 | 1.12 | 0.70 | 0.43 |
| **St.Dev** | **0.33** | **0.20** | **0.23** | **0.19** |  | 2.67 | 1.53 | 1.09 | 0.63 |
|  | | | | |  | 1.96 | 0.80 | 0.67 | 0.36 |
|  |  |  |  |  |  | 3.04 | 1.54 | 0.91 | 0.57 |
|  |  |  |  |  |  | 2.18 | 0.76 | 0.38 | 0.29 |
|  |  |  |  |  |  | 3.08 | 1.64 | 1.01 | 0.68 |
|  |  |  |  |  |  | 2.49 | 1.16 | 1.03 | 0.54 |
|  |  |  |  |  |  | 1.70 | 0.89 | 0.72 | 0.34 |
|  |  |  |  |  |  | 2.53 | 1.43 | 1.08 | 0.74 |
|  |  |  |  |  |  | 2.64 | 1.27 | 1.13 | 0.74 |
|  |  |  |  |  |  | 3.04 | 1.71 | 1.68 | 1.24 |
|  |  |  |  |  |  | 3.27 | 1.17 | 1.01 | 0.60 |
|  |  |  |  |  |  | 2.49 | 0.82 | 0.86 | 0.39 |
|  |  |  |  |  |  | 2.29 | 1.37 | 1.15 | 0.81 |
|  |  |  |  |  |  | 1.75 | 0.81 | 0.51 | 0.43 |
|  |  |  |  |  |  | 1.62 | 0.64 | 0.64 | 0.62 |
|  |  |  |  |  | **Avg** | **2.46** | **1.14** | **0.94** | **0.63** |
|  |  |  |  |  | **St.Dev** | **0.61** | **0.38** | **0.33** | **0.25** |

**Tb.Th (mm)**

|  | **8 weeks** | **12 weeks** | **16 weeks** | **20 weeks** |  | **8 weeks** | **12 weeks** | **16 weeks** | **20 weeks** |
| --- | --- | --- | --- | --- | --- | --- | --- | --- | --- |
| **Placebo** | 0.103 | 0.104 | 0.104 | 0.104 | **LPS** | 0.084 | 0.085 | 0.095 | 0.088 |
|  | 0.096 | 0.098 | 0.102 | 0.103 |  | 0.084 | 0.088 | 0.092 | 0.095 |
|  | 0.095 | 0.094 | 0.098 | 0.092 |  | 0.084 | 0.079 | 0.091 | 0.085 |
|  | 0.084 | 0.088 | 0.071 | 0.057 |  | 0.093 | 0.100 | 0.107 | 0.106 |
|  | 0.100 | 0.089 | 0.081 | 0.082 |  | 0.086 | 0.083 | 0.090 | 0.094 |
|  | 0.090 | 0.103 | 0.108 | 0.101 |  | 0.116 | 0.109 | 0.112 | 0.109 |
|  | 0.083 | 0.093 | 0.099 | 0.095 |  | 0.096 | 0.093 | 0.092 | 0.088 |
|  | 0.094 | 0.096 | 0.100 | 0.100 |  | 0.093 | 0.090 | 0.094 | 0.098 |
|  | 0.086 | 0.094 | 0.094 | 0.086 |  | 0.082 | 0.091 | 0.098 | 0.104 |
|  | 0.087 | 0.088 | 0.081 | 0.075 |  | 0.093 | 0.087 | 0.094 | 0.090 |
|  | 0.085 | 0.083 | 0.087 | 0.084 |  | 0.092 | 0.087 | 0.089 | 0.083 |
| **Avg** | **0.091** | **0.094** | **0.093** | **0.089** |  | 0.091 | 0.086 | 0.086 | 0.098 |
| **St.Dev** | **0.007** | **0.007** | **0.011** | **0.014** |  | 0.093 | 0.094 | 0.087 | 0.090 |
|  | | | | |  | 0.089 | 0.092 | 0.090 | 0.088 |
|  |  |  |  |  |  | 0.087 | 0.095 | 0.098 | 0.093 |
|  |  |  |  |  |  | 0.084 | 0.080 | 0.081 | 0.092 |
|  |  |  |  |  |  | 0.103 | 0.091 | 0.095 | 0.090 |
|  |  |  |  |  |  | 0.091 | 0.087 | 0.094 | 0.084 |
|  |  |  |  |  |  | 0.087 | 0.090 | 0.103 | 0.101 |
|  |  |  |  |  |  | 0.086 | 0.094 | 0.103 | 0.107 |
|  |  |  |  |  |  | 0.086 | 0.088 | 0.092 | 0.089 |
|  |  |  |  |  |  | 0.088 | 0.092 | 0.096 | 0.092 |
|  |  |  |  |  |  | 0.100 | 0.084 | 0.077 | 0.085 |
|  |  |  |  |  |  | 0.087 | 0.082 | 0.084 | 0.083 |
|  |  |  |  |  |  | 0.093 | 0.092 | 0.096 | 0.090 |
|  |  |  |  |  |  | 0.081 | 0.086 | 0.099 | 0.110 |
|  |  |  |  |  |  | 0.078 | 0.076 | 0.083 | 0.085 |
|  |  |  |  |  | **Avg** | **0.090** | **0.089** | **0.093** | **0.093** |
|  |  |  |  |  | **St.Dev** | **0.008** | **0.007** | **0.008** | **0.008** |

**Tb.Sp (mm)**

|  | **8 weeks** | **12 weeks** | **16 weeks** | **20 weeks** |  | **8 weeks** | **12 weeks** | **16 weeks** | **20 weeks** |
| --- | --- | --- | --- | --- | --- | --- | --- | --- | --- |
| **Placebo** | 0.394 | 0.455 | 0.504 | 0.537 | **LPS** | 0.342 | 0.560 | 0.558 | 0.558 |
|  | 0.325 | 0.400 | 0.465 | 0.525 |  | 0.323 | 0.523 | 0.552 | 0.562 |
|  | 0.281 | 0.432 | 0.491 | 0.537 |  | 0.370 | 0.504 | 0.549 | 0.554 |
|  | 0.236 | 0.364 | 0.406 | 0.463 |  | 0.352 | 0.407 | 0.466 | 0.505 |
|  | 0.294 | 0.449 | 0.548 | 0.567 |  | 0.305 | 0.422 | 0.481 | 0.519 |
|  | 0.318 | 0.435 | 0.475 | 0.527 |  | 0.237 | 0.417 | 0.423 | 0.471 |
|  | 0.357 | 0.447 | 0.531 | 0.563 |  | 0.158 | 0.322 | 0.358 | 0.465 |
|  | 0.275 | 0.432 | 0.486 | 0.493 |  | 0.336 | 0.459 | 0.502 | 0.534 |
|  | 0.345 | 0.401 | 0.461 | 0.521 |  | 0.262 | 0.428 | 0.472 | 0.518 |
|  | 0.281 | 0.407 | 0.473 | 0.510 |  | 0.321 | 0.494 | 0.543 | 0.546 |
|  | 0.336 | 0.453 | 0.505 | 0.500 |  | 0.312 | 0.383 | 0.418 | 0.446 |
| **Avg** | **0.313** | **0.425** | **0.486** | **0.522** |  | 0.334 | 0.493 | 0.549 | 0.573 |
| **St.Dev** | **0.045** | **0.029** | **0.038** | **0.030** |  | 0.235 | 0.394 | 0.434 | 0.482 |
|  | | | | |  | 0.421 | 0.535 | 0.563 | 0.571 |
|  |  |  |  |  |  | 0.256 | 0.412 | 0.476 | 0.517 |
|  |  |  |  |  |  | 0.265 | 0.510 | 0.548 | 0.567 |
|  |  |  |  |  |  | 0.244 | 0.386 | 0.449 | 0.509 |
|  |  |  |  |  |  | 0.280 | 0.456 | 0.481 | 0.504 |
|  |  |  |  |  |  | 0.309 | 0.475 | 0.488 | 0.542 |
|  |  |  |  |  |  | 0.296 | 0.453 | 0.525 | 0.539 |
|  |  |  |  |  |  | 0.238 | 0.425 | 0.444 | 0.506 |
|  |  |  |  |  |  | 0.207 | 0.341 | 0.398 | 0.433 |
|  |  |  |  |  |  | 0.238 | 0.408 | 0.455 | 0.532 |
|  |  |  |  |  |  | 0.341 | 0.460 | 0.509 | 0.545 |
|  |  |  |  |  |  | 0.394 | 0.428 | 0.445 | 0.465 |
|  |  |  |  |  |  | 0.331 | 0.548 | 0.568 | 0.561 |
|  |  |  |  |  |  | 0.295 | 0.538 | 0.526 | 0.533 |
|  |  |  |  |  | **Avg** | **0.296** | **0.451** | **0.488** | **0.521** |
|  |  |  |  |  | **St.Dev** | **0.059** | **0.062** | **0.056** | **0.039** |

**DA (no unit)**

|  | **8 weeks** | **12 weeks** | **16 weeks** | **20 weeks** |  | **8 weeks** | **12 weeks** | **16 weeks** | **20 weeks** |
| --- | --- | --- | --- | --- | --- | --- | --- | --- | --- |
| **Placebo** | 2.17 | 1.91 | 2.08 | 2.07 | **LPS** | 2.25 | 1.98 | 2.03 | 2.06 |
|  | 1.76 | 1.74 | 1.87 | 1.57 |  | 1.98 | 2.02 | 2.08 | 2.08 |
|  | 2.11 | 2.36 | 1.93 | 1.65 |  | 2.03 | 2.09 | 1.81 | 1.97 |
|  | 1.83 | 1.80 | 1.65 | 1.46 |  | 2.00 | 1.74 | 1.75 | 1.52 |
|  | 1.80 | 2.13 | 2.12 | 2.11 |  | 2.09 | 2.02 | 1.96 | 1.81 |
|  | 2.24 | 2.20 | 1.93 | 2.19 |  | 1.63 | 1.88 | 2.00 | 1.98 |
|  | 2.08 | 1.96 | 1.97 | 1.85 |  | 1.69 | 1.79 | 1.65 | 1.44 |
|  | 1.93 | 1.81 | 1.95 | 2.01 |  | 2.08 | 2.15 | 2.35 | 2.23 |
|  | 1.85 | 1.91 | 2.16 | 2.06 |  | 1.87 | 2.16 | 1.77 | 2.07 |
|  | 2.03 | 1.86 | 2.16 | 2.09 |  | 1.75 | 1.94 | 1.92 | 1.90 |
|  | 1.92 | 1.87 | 2.02 | 1.82 |  | 1.84 | 2.13 | 1.92 | 1.83 |
| **Avg** | **1.98** | **1.96** | **1.99** | **1.90** |  | 1.94 | 2.40 | 2.49 | 1.99 |
| **St.Dev** | **0.16** | **0.19** | **0.15** | **0.25** |  | 2.26 | 2.14 | 1.71 | 1.59 |
|  | | | | |  | 1.86 | 1.97 | 1.94 | 2.09 |
|  |  |  |  |  |  | 2.00 | 2.14 | 2.42 | 1.76 |
|  |  |  |  |  |  | 2.09 | 1.75 | 2.33 | 2.28 |
|  |  |  |  |  |  | 1.99 | 2.00 | 1.94 | 2.18 |
|  |  |  |  |  |  | 1.83 | 1.74 | 1.62 | 1.79 |
|  |  |  |  |  |  | 1.95 | 2.20 | 1.85 | 1.77 |
|  |  |  |  |  |  | 1.75 | 1.63 | 1.90 | 1.79 |
|  |  |  |  |  |  | 2.23 | 2.13 | 2.00 | 2.04 |
|  |  |  |  |  |  | 2.03 | 1.88 | 1.81 | 1.66 |
|  |  |  |  |  |  | 1.93 | 1.99 | 2.12 | 2.14 |
|  |  |  |  |  |  | 2.16 | 1.78 | 1.97 | 2.21 |
|  |  |  |  |  |  | 1.90 | 1.92 | 1.87 | 1.95 |
|  |  |  |  |  |  | 1.88 | 1.72 | 1.55 | 1.66 |
|  |  |  |  |  |  | 1.96 | 2.14 | 1.79 | 1.88 |
|  |  |  |  |  | **Avg** | **1.96** | **1.98** | **1.95** | **1.91** |
|  |  |  |  |  | **St.Dev** | **0.16** | **0.18** | **0.24** | **0.22** |

**SMI**

|  | **8 weeks** | **12 weeks** | **16 weeks** | **20 weeks** |  | **8 weeks** | **12 weeks** | **16 weeks** | **20 weeks** |
| --- | --- | --- | --- | --- | --- | --- | --- | --- | --- |
| **Placebo** | 2.20 | 2.38 | 2.60 | 2.66 | **LPS** | 2.24 | 2.77 | 2.89 | 2.97 |
|  | 2.04 | 2.81 | 2.94 | 2.83 |  | 2.42 | 2.75 | 2.73 | 2.63 |
|  | 1.99 | 2.30 | 2.57 | 2.75 |  | 2.35 | 2.63 | 2.81 | 2.91 |
|  | 2.22 | 2.71 | 2.75 | 2.77 |  | 2.07 | 2.69 | 2.59 | 2.45 |
|  | 2.02 | 2.70 | 3.00 | 3.15 |  | 2.10 | 2.65 | 2.69 | 2.75 |
|  | 2.42 | 2.85 | 2.71 | 2.83 |  | 1.51 | 2.34 | 2.13 | 2.62 |
|  | 2.25 | 2.39 | 2.43 | 2.57 |  | 1.36 | 2.62 | 2.82 | 3.02 |
|  | 2.49 | 2.41 | 2.70 | 2.96 |  | 2.07 | 2.38 | 2.29 | 2.78 |
|  | 2.30 | 2.47 | 2.69 | 2.67 |  | 2.15 | 2.29 | 2.61 | 2.72 |
|  | 2.42 | 2.58 | 2.93 | 3.18 |  | 1.99 | 2.56 | 2.65 | 2.70 |
|  | 2.47 | 2.62 | 3.09 | 2.96 |  | 1.88 | 2.43 | 2.49 | 2.71 |
| **Avg** | **2.26** | **2.56** | **2.76** | **2.85** |  | 2.14 | 2.35 | 2.57 | 3.04 |
| **St.Dev** | **0.18** | **0.19** | **0.20** | **0.20** |  | 2.13 | 2.49 | 2.56 | 2.97 |
|  | | | | |  | 2.17 | 2.81 | 2.61 | 2.43 |
|  |  |  |  |  |  | 1.75 | 2.44 | 2.65 | 2.89 |
|  |  |  |  |  |  | 2.51 | 2.75 | 2.86 | 3.13 |
|  |  |  |  |  |  | 1.83 | 2.43 | 2.78 | 2.82 |
|  |  |  |  |  |  | 2.06 | 2.52 | 2.50 | 2.73 |
|  |  |  |  |  |  | 2.43 | 2.70 | 2.85 | 3.25 |
|  |  |  |  |  |  | 2.10 | 2.24 | 2.36 | 2.95 |
|  |  |  |  |  |  | 2.22 | 2.65 | 2.39 | 2.77 |
|  |  |  |  |  |  | 1.93 | 2.45 | 2.30 | 2.46 |
|  |  |  |  |  |  | 1.59 | 2.65 | 2.69 | 2.96 |
|  |  |  |  |  |  | 1.84 | 2.62 | 2.57 | 3.31 |
|  |  |  |  |  |  | 1.87 | 2.52 | 2.74 | 2.92 |
|  |  |  |  |  |  | 2.41 | 2.51 | 2.85 | 2.96 |
|  |  |  |  |  |  | 2.65 | 2.59 | 2.74 | 2.55 |
|  |  |  |  |  | **Avg** | **2.07** | **2.55** | **2.62** | **2.83** |
|  |  |  |  |  | **St.Dev** | **0.30** | **0.15** | **0.20** | **0.23** |

**vBMD (g/cm^3^)**

|  | **8 weeks** | **12 weeks** | **16 weeks** | **20 weeks** |  | **8 weeks** | **12 weeks** | **16 weeks** | **20 weeks** |
| --- | --- | --- | --- | --- | --- | --- | --- | --- | --- |
| **Placebo** | 0.226 | 0.195 | 0.151 | 0.121 | **LPS** | 0.203 | 0.120 | 0.129 | 0.125 |
|  | 0.248 | 0.190 | 0.166 | 0.141 |  | 0.203 | 0.138 | 0.136 | 0.129 |
|  | 0.253 | 0.193 | 0.178 | 0.153 |  | 0.193 | 0.126 | 0.137 | 0.124 |
|  | 0.241 | 0.187 | 0.157 | 0.136 |  | 0.226 | 0.188 | 0.195 | 0.185 |
|  | 0.259 | 0.166 | 0.120 | 0.116 |  | 0.214 | 0.155 | 0.147 | 0.138 |
|  | 0.200 | 0.159 | 0.145 | 0.107 |  | 0.330 | 0.231 | 0.225 | 0.190 |
|  | 0.208 | 0.178 | 0.153 | 0.125 |  | 0.323 | 0.199 | 0.184 | 0.157 |
|  | 0.221 | 0.186 | 0.174 | 0.157 |  | 0.223 | 0.177 | 0.157 | 0.142 |
|  | 0.216 | 0.192 | 0.170 | 0.141 |  | 0.233 | 0.178 | 0.169 | 0.153 |
|  | 0.215 | 0.180 | 0.147 | 0.128 |  | 0.242 | 0.150 | 0.150 | 0.138 |
|  | 0.203 | 0.162 | 0.138 | 0.125 |  | 0.250 | 0.186 | 0.179 | 0.158 |
| **Avg** | **0.226** | **0.181** | **0.155** | **0.132** |  | 0.225 | 0.159 | 0.145 | 0.134 |
| **St.Dev** | **0.021** | **0.013** | **0.017** | **0.015** |  | 0.251 | 0.192 | 0.166 | 0.148 |
|  | | | | |  | 0.205 | 0.149 | 0.142 | 0.125 |
|  |  |  |  |  |  | 0.258 | 0.190 | 0.159 | 0.133 |
|  |  |  |  |  |  | 0.218 | 0.142 | 0.128 | 0.123 |
|  |  |  |  |  |  | 0.283 | 0.198 | 0.172 | 0.153 |
|  |  |  |  |  |  | 0.237 | 0.164 | 0.166 | 0.136 |
|  |  |  |  |  |  | 0.200 | 0.149 | 0.150 | 0.127 |
|  |  |  |  |  |  | 0.236 | 0.186 | 0.174 | 0.159 |
|  |  |  |  |  |  | 0.240 | 0.174 | 0.163 | 0.144 |
|  |  |  |  |  |  | 0.257 | 0.200 | 0.200 | 0.177 |
|  |  |  |  |  |  | 0.287 | 0.167 | 0.158 | 0.142 |
|  |  |  |  |  |  | 0.231 | 0.148 | 0.149 | 0.128 |
|  |  |  |  |  |  | 0.224 | 0.177 | 0.174 | 0.154 |
|  |  |  |  |  |  | 0.196 | 0.148 | 0.141 | 0.142 |
|  |  |  |  |  |  | 0.194 | 0.137 | 0.142 | 0.148 |
|  |  |  |  |  | **Avg** | **0.236** | **0.168** | **0.161** | **0.145** |
|  |  |  |  |  | **St.Dev** | **0.036** | **0.026** | **0.023** | **0.018** |
