# Supplementary material for: Trabecular and cortical bone are unaltered in response to chronic lipopolysaccharide exposure via osmotic pumps in male and female CD-1 mice
Source: PLoS One. 2021 Feb 5;16(2):e0243933. doi: 10.1371/journal.pone.0243933 (PMC7864436; doi:10.1371/journal.pone.0243933)
Supplement: S5 Table — (DOCX) [file pone.0243933.s005.docx]

**Cortical Bone**

- Each row represents an individual animal and columns represent timepoints

**Males**

**Ct.Ar/T.Ar (%)**

|  | **8 weeks** | **12 weeks** | **16 weeks** | **20 weeks** |  | **8 weeks** | **12 weeks** | **16 weeks** | **20 weeks** |
| --- | --- | --- | --- | --- | --- | --- | --- | --- | --- |
| **Placebo** | 66.54 | 68.09 | 68.49 | 69.06 | **LPS** | 68.91 | 69.24 | 69.14 | 69.10 |
|  | 62.59 | 64.44 | 64.56 | 64.73 |  | 70.95 | 71.18 | 71.71 | 71.67 |
|  | 67.05 | 69.08 | 70.75 | 71.17 |  | 68.83 | 68.65 | 69.71 | 69.42 |
|  | 63.43 | 65.45 | 67.00 | 66.78 |  | 73.01 | 73.59 | 73.98 | 74.08 |
|  | 65.07 | 64.97 | 66.40 | 68.53 |  | 69.97 | 68.74 | 68.49 | 67.46 |
|  | 64.86 | 65.07 | 65.55 | 67.08 |  | 64.01 | 64.48 | 66.51 | 67.95 |
|  | 63.06 | 63.82 | 66.70 | 66.13 |  | 67.50 | 67.13 | 68.11 | 69.77 |
|  | 68.30 | 68.12 | 68.19 | 68.69 |  | 62.31 | 61.72 | 62.00 | 64.67 |
|  | 64.39 | 62.97 | 63.48 | 65.48 |  | 66.19 | 66.19 | 67.43 | 69.12 |
|  | 66.63 | 64.45 | 65.42 | 66.52 |  | 62.29 | 63.44 | 62.95 | 63.23 |
|  | 64.81 | 66.04 | 65.60 | 67.93 |  | 64.10 | 65.97 | 66.67 | 66.79 |
|  | 66.91 | 65.75 | 66.04 | 66.41 |  | 62.17 | 63.92 | 65.44 | 65.73 |
| **Avg** | **65.30** | **65.69** | **66.52** | **67.37** |  | 64.31 | 64.30 | 62.96 | 62.70 |
| **St.Dev** | **1.79** | **1.87** | **1.93** | **1.78** |  | 65.23 | 64.63 | 64.77 | 64.27 |
|  | | | | |  | 61.89 | 61.00 | 60.54 | 61.03 |
|  |  |  |  |  |  | 66.15 | 66.83 | 67.91 | 69.24 |
|  |  |  |  |  |  | 67.34 | 68.78 | 68.50 | 70.43 |
|  |  |  |  |  |  | 66.47 | 65.83 | 67.20 | 68.29 |
|  |  |  |  |  |  | 64.53 | 64.98 | 69.20 | 67.66 |
|  |  |  |  |  |  | 61.74 | 61.64 | 61.89 | 62.76 |
|  |  |  |  |  |  | 64.93 | 65.92 | 66.58 | 66.50 |
|  |  |  |  |  |  | 63.03 | 61.96 | 62.40 | 61.88 |
|  |  |  |  |  |  | 65.17 | 64.51 | 65.02 | 64.50 |
|  |  |  |  |  |  | 67.39 | 68.15 | 68.98 | 69.04 |
|  |  |  |  |  |  | 60.36 | 60.03 | 61.22 | 62.27 |
|  |  |  |  |  |  | 69.89 | 67.90 | 67.04 | 67.80 |
|  |  |  |  |  |  | 68.80 | 68.38 | 69.94 | 70.81 |
|  |  |  |  |  |  | 64.83 | 65.16 | 66.86 | 68.71 |
|  |  |  |  |  |  | 62.27 | 63.03 | 64.56 | 66.85 |
|  |  |  |  |  |  | 63.17 | 63.20 | 64.84 | 65.75 |
|  |  |  |  |  | **Avg** | **65.59** | **65.68** | **66.42** | **66.98** |
|  |  |  |  |  | **St.Dev** | **3.14** | **3.12** | **3.21** | **3.19** |

**Ct.Th (mm)**

|  | **8 weeks** | **12 weeks** | **16 weeks** | **20 weeks** |  | **8 weeks** | **12 weeks** | **16 weeks** | **20 weeks** |
| --- | --- | --- | --- | --- | --- | --- | --- | --- | --- |
| **Placebo** | 0.230 | 0.238 | 0.236 | 0.241 | **LPS** | 0.227 | 0.235 | 0.234 | 0.239 |
|  | 0.212 | 0.222 | 0.223 | 0.220 |  | 0.258 | 0.261 | 0.264 | 0.268 |
|  | 0.232 | 0.242 | 0.252 | 0.248 |  | 0.241 | 0.246 | 0.249 | 0.249 |
|  | 0.214 | 0.225 | 0.237 | 0.235 |  | 0.268 | 0.271 | 0.280 | 0.277 |
|  | 0.221 | 0.221 | 0.230 | 0.243 |  | 0.264 | 0.267 | 0.266 | 0.262 |
|  | 0.243 | 0.249 | 0.246 | 0.260 |  | 0.241 | 0.246 | 0.259 | 0.266 |
|  | 0.231 | 0.236 | 0.243 | 0.246 |  | 0.257 | 0.258 | 0.260 | 0.266 |
|  | 0.258 | 0.254 | 0.249 | 0.259 |  | 0.226 | 0.226 | 0.228 | 0.242 |
|  | 0.230 | 0.224 | 0.220 | 0.235 |  | 0.254 | 0.255 | 0.262 | 0.277 |
|  | 0.245 | 0.240 | 0.241 | 0.248 |  | 0.225 | 0.234 | 0.234 | 0.236 |
|  | 0.217 | 0.221 | 0.216 | 0.234 |  | 0.240 | 0.246 | 0.254 | 0.254 |
|  | 0.253 | 0.243 | 0.253 | 0.248 |  | 0.204 | 0.211 | 0.220 | 0.223 |
| **Avg** | **0.232** | **0.234** | **0.237** | **0.243** |  | 0.248 | 0.255 | 0.251 | 0.249 |
| **St.Dev** | **0.015** | **0.012** | **0.012** | **0.011** |  | 0.238 | 0.234 | 0.237 | 0.236 |
|  | | | | |  | 0.223 | 0.216 | 0.219 | 0.221 |
|  |  |  |  |  |  | 0.229 | 0.236 | 0.239 | 0.247 |
|  |  |  |  |  |  | 0.239 | 0.250 | 0.245 | 0.260 |
|  |  |  |  |  |  | 0.249 | 0.247 | 0.255 | 0.260 |
|  |  |  |  |  |  | 0.237 | 0.243 | 0.271 | 0.259 |
|  |  |  |  |  |  | 0.219 | 0.220 | 0.220 | 0.229 |
|  |  |  |  |  |  | 0.227 | 0.236 | 0.233 | 0.235 |
|  |  |  |  |  |  | 0.225 | 0.219 | 0.229 | 0.223 |
|  |  |  |  |  |  | 0.235 | 0.235 | 0.239 | 0.235 |
|  |  |  |  |  |  | 0.227 | 0.236 | 0.236 | 0.244 |
|  |  |  |  |  |  | 0.227 | 0.222 | 0.225 | 0.230 |
|  |  |  |  |  |  | 0.259 | 0.252 | 0.243 | 0.249 |
|  |  |  |  |  |  | 0.249 | 0.253 | 0.255 | 0.264 |
|  |  |  |  |  |  | 0.231 | 0.237 | 0.237 | 0.256 |
|  |  |  |  |  |  | 0.237 | 0.245 | 0.251 | 0.264 |
|  |  |  |  |  |  | 0.233 | 0.229 | 0.238 | 0.246 |
|  |  |  |  |  | **Avg** | **0.238** | **0.241** | **0.244** | **0.249** |
|  |  |  |  |  | **St.Dev** | **0.015** | **0.015** | **0.016** | **0.016** |

**Ps.Pm (mm)**

|  | **8 weeks** | **12 weeks** | **16 weeks** | **20 weeks** |  | **8 weeks** | **12 weeks** | **16 weeks** | **20 weeks** |
| --- | --- | --- | --- | --- | --- | --- | --- | --- | --- |
| **Placebo** | 6.722 | 6.680 | 6.696 | 6.658 | **LPS** | 6.104 | 6.361 | 6.304 | 6.501 |
|  | 7.112 | 7.212 | 7.440 | 7.246 |  | 6.791 | 6.899 | 6.977 | 7.052 |
|  | 6.732 | 6.669 | 6.795 | 6.566 |  | 6.838 | 6.964 | 6.934 | 7.121 |
|  | 7.236 | 7.277 | 7.417 | 7.340 |  | 6.718 | 6.631 | 6.778 | 6.689 |
|  | 6.972 | 6.984 | 7.066 | 7.127 |  | 7.247 | 7.523 | 7.501 | 7.721 |
|  | 7.462 | 7.456 | 7.359 | 7.709 |  | 8.017 | 8.079 | 8.173 | 7.917 |
|  | 7.808 | 7.927 | 7.168 | 8.093 |  | 7.597 | 7.782 | 7.731 | 7.569 |
|  | 7.511 | 7.337 | 7.377 | 7.518 |  | 7.531 | 7.856 | 7.802 | 7.893 |
|  | 7.394 | 7.629 | 7.500 | 7.504 |  | 7.416 | 7.572 | 7.549 | 7.667 |
|  | 7.402 | 7.829 | 7.597 | 7.742 |  | 7.664 | 7.773 | 7.841 | 7.881 |
|  | 7.043 | 6.906 | 7.044 | 7.251 |  | 8.404 | 7.882 | 8.245 | 8.030 |
|  | 7.705 | 7.949 | 8.155 | 7.981 |  | 6.864 | 6.822 | 6.912 | 6.852 |
| **Avg** | **7.258** | **7.321** | **7.301** | **7.395** |  | 7.508 | 7.741 | 7.930 | 8.120 |
| **St.Dev** | **0.352** | **0.454** | **0.389** | **0.470** |  | 7.390 | 7.288 | 7.325 | 7.578 |
|  | | | | |  | 7.506 | 7.557 | 7.796 | 7.816 |
|  |  |  |  |  |  | 6.919 | 7.026 | 7.051 | 7.073 |
|  |  |  |  |  |  | 7.024 | 6.970 | 7.421 | 7.399 |
|  |  |  |  |  |  | 7.526 | 8.146 | 8.101 | 7.974 |
|  |  |  |  |  |  | 7.401 | 7.692 | 8.066 | 8.229 |
|  |  |  |  |  |  | 7.215 | 7.324 | 7.275 | 7.552 |
|  |  |  |  |  |  | 7.530 | 7.547 | 7.574 | 7.617 |
|  |  |  |  |  |  | 7.370 | 7.298 | 7.685 | 7.548 |
|  |  |  |  |  |  | 7.283 | 7.420 | 7.531 | 7.647 |
|  |  |  |  |  |  | 6.645 | 6.883 | 6.746 | 7.100 |
|  |  |  |  |  |  | 7.914 | 7.882 | 7.802 | 7.991 |
|  |  |  |  |  |  | 7.076 | 7.323 | 7.329 | 7.375 |
|  |  |  |  |  |  | 7.048 | 7.292 | 7.188 | 7.233 |
|  |  |  |  |  |  | 7.317 | 7.409 | 7.195 | 7.506 |
|  |  |  |  |  |  | 8.052 | 8.308 | 8.105 | 8.000 |
|  |  |  |  |  |  | 7.876 | 7.755 | 7.780 | 8.052 |
|  |  |  |  |  | **Avg** | **7.326** | **7.433** | **7.488** | **7.557** |
|  |  |  |  |  | **St.Dev** | **0.480** | **0.465** | **0.483** | **0.443** |

**Ec.Pm (mm)**

|  | **8 weeks** | **12 weeks** | **16 weeks** | **20 weeks** |  | **8 weeks** | **12 weeks** | **16 weeks** | **20 weeks** |
| --- | --- | --- | --- | --- | --- | --- | --- | --- | --- |
| **Placebo** | 2.461 | 2.403 | 2.378 | 2.347 | **LPS** | 2.241 | 2.332 | 2.322 | 2.371 |
|  | 2.762 | 2.748 | 2.828 | 2.722 |  | 2.409 | 2.438 | 2.451 | 2.469 |
|  | 2.438 | 2.343 | 2.318 | 2.252 |  | 2.472 | 2.494 | 2.435 | 2.485 |
|  | 2.856 | 2.779 | 2.734 | 2.730 |  | 2.322 | 2.236 | 2.265 | 2.266 |
|  | 2.675 | 2.686 | 2.665 | 2.612 |  | 2.665 | 2.770 | 2.751 | 2.871 |
|  | 2.752 | 2.757 | 2.709 | 2.773 |  | 3.175 | 3.147 | 3.040 | 2.911 |
|  | 3.068 | 3.042 | 2.677 | 2.999 |  | 2.799 | 2.846 | 2.750 | 2.643 |
|  | 2.808 | 2.732 | 2.711 | 2.733 |  | 2.925 | 3.040 | 2.997 | 2.958 |
|  | 2.783 | 2.944 | 2.813 | 2.789 |  | 2.766 | 2.815 | 2.735 | 2.726 |
|  | 2.767 | 3.008 | 2.875 | 2.897 |  | 2.986 | 2.948 | 2.974 | 2.973 |
|  | 2.673 | 2.569 | 2.627 | 2.613 |  | 3.312 | 3.025 | 3.149 | 3.042 |
|  | 2.874 | 2.998 | 2.981 | 2.960 |  | 2.675 | 2.600 | 2.570 | 2.526 |
| **Avg** | **2.743** | **2.751** | **2.693** | **2.702** |  | 2.789 | 2.891 | 2.981 | 3.035 |
| **St.Dev** | **0.172** | **0.229** | **0.190** | **0.224** |  | 2.835 | 2.773 | 2.774 | 2.863 |
|  | | | | |  | 2.956 | 2.947 | 3.010 | 3.002 |
|  |  |  |  |  |  | 2.615 | 2.595 | 2.545 | 2.509 |
|  |  |  |  |  |  | 2.640 | 2.535 | 2.737 | 2.629 |
|  |  |  |  |  |  | 2.766 | 3.034 | 2.939 | 2.884 |
|  |  |  |  |  |  | 2.792 | 2.845 | 2.816 | 2.928 |
|  |  |  |  |  |  | 2.818 | 2.865 | 2.817 | 2.908 |
|  |  |  |  |  |  | 2.830 | 2.775 | 2.721 | 2.754 |
|  |  |  |  |  |  | 2.841 | 2.818 | 2.944 | 2.921 |
|  |  |  |  |  |  | 2.733 | 2.767 | 2.781 | 2.827 |
|  |  |  |  |  |  | 2.428 | 2.479 | 2.420 | 2.521 |
|  |  |  |  |  |  | 3.044 | 3.041 | 2.971 | 3.016 |
|  |  |  |  |  |  | 2.567 | 2.688 | 2.687 | 2.689 |
|  |  |  |  |  |  | 2.592 | 2.667 | 2.574 | 2.565 |
|  |  |  |  |  |  | 2.789 | 2.774 | 2.641 | 2.694 |
|  |  |  |  |  |  | 3.153 | 3.187 | 3.050 | 2.954 |
|  |  |  |  |  |  | 3.015 | 2.956 | 2.877 | 2.942 |
|  |  |  |  |  | **Avg** | **2.765** | **2.778** | **2.757** | **2.763** |
|  |  |  |  |  | **St.Dev** | **0.250** | **0.236** | **0.231** | **0.219** |

**Tt.Ar(mm^2^)**

|  | **8 weeks** | **12 weeks** | **16 weeks** | **20 weeks** |  | **8 weeks** | **12 weeks** | **16 weeks** | **20 weeks** |
| --- | --- | --- | --- | --- | --- | --- | --- | --- | --- |
| **Placebo** | 1.163 | 1.165 | 1.153 | 1.162 | **LPS** | 1.004 | 1.081 | 1.067 | 1.123 |
|  | 1.202 | 1.240 | 1.286 | 1.233 |  | 1.236 | 1.264 | 1.284 | 1.317 |
|  | 1.167 | 1.170 | 1.209 | 1.143 |  | 1.196 | 1.249 | 1.241 | 1.278 |
|  | 1.219 | 1.250 | 1.313 | 1.293 |  | 1.232 | 1.222 | 1.282 | 1.249 |
|  | 1.185 | 1.190 | 1.224 | 1.266 |  | 1.369 | 1.463 | 1.456 | 1.498 |
|  | 1.397 | 1.429 | 1.381 | 1.496 |  | 1.508 | 1.542 | 1.591 | 1.548 |
|  | 1.427 | 1.463 | 1.304 | 1.507 |  | 1.448 | 1.496 | 1.475 | 1.445 |
|  | 1.420 | 1.370 | 1.347 | 1.416 |  | 1.364 | 1.441 | 1.434 | 1.476 |
|  | 1.323 | 1.354 | 1.302 | 1.344 |  | 1.422 | 1.458 | 1.469 | 1.534 |
|  | 1.364 | 1.456 | 1.399 | 1.445 |  | 1.383 | 1.432 | 1.458 | 1.471 |
|  | 1.181 | 1.154 | 1.162 | 1.249 |  | 1.574 | 1.472 | 1.568 | 1.528 |
|  | 1.457 | 1.466 | 1.563 | 1.491 |  | 1.125 | 1.126 | 1.161 | 1.161 |
| **Avg** | **1.292** | **1.309** | **1.304** | **1.337** |  | 1.446 | 1.535 | 1.582 | 1.614 |
| **St.Dev** | **0.116** | **0.127** | **0.114** | **0.131** |  | 1.347 | 1.317 | 1.338 | 1.388 |
|  | | | | |  | 1.355 | 1.339 | 1.413 | 1.413 |
|  |  |  |  |  |  | 1.197 | 1.242 | 1.243 | 1.261 |
|  |  |  |  |  |  | 1.246 | 1.265 | 1.329 | 1.366 |
|  |  |  |  |  |  | 1.411 | 1.530 | 1.538 | 1.520 |
|  |  |  |  |  |  | 1.358 | 1.439 | 1.577 | 1.573 |
|  |  |  |  |  |  | 1.277 | 1.307 | 1.294 | 1.375 |
|  |  |  |  |  |  | 1.319 | 1.349 | 1.325 | 1.347 |
|  |  |  |  |  |  | 1.317 | 1.292 | 1.409 | 1.361 |
|  |  |  |  |  |  | 1.311 | 1.351 | 1.382 | 1.395 |
|  |  |  |  |  |  | 1.120 | 1.190 | 1.156 | 1.253 |
|  |  |  |  |  |  | 1.488 | 1.455 | 1.432 | 1.475 |
|  |  |  |  |  |  | 1.313 | 1.358 | 1.328 | 1.356 |
|  |  |  |  |  |  | 1.276 | 1.350 | 1.312 | 1.346 |
|  |  |  |  |  |  | 1.304 | 1.347 | 1.276 | 1.397 |
|  |  |  |  |  |  | 1.535 | 1.617 | 1.578 | 1.577 |
|  |  |  |  |  |  | 1.449 | 1.404 | 1.431 | 1.505 |
|  |  |  |  |  | **Avg** | **1.331** | **1.364** | **1.381** | **1.405** |
|  |  |  |  |  | **St.Dev** | **0.129** | **0.128** | **0.138** | **0.124** |

**Ma.Ar (mm^2^)**

|  | **8 weeks** | **12 weeks** | **16 weeks** | **20 weeks** |  | **8 weeks** | **12 weeks** | **16 weeks** | **20 weeks** |
| --- | --- | --- | --- | --- | --- | --- | --- | --- | --- |
| **Placebo** | 0.389 | 0.372 | 0.363 | 0.360 | **LPS** | 0.312 | 0.332 | 0.329 | 0.347 |
|  | 0.450 | 0.441 | 0.456 | 0.435 |  | 0.359 | 0.364 | 0.363 | 0.373 |
|  | 0.384 | 0.362 | 0.354 | 0.329 |  | 0.373 | 0.392 | 0.376 | 0.391 |
|  | 0.446 | 0.432 | 0.433 | 0.430 |  | 0.333 | 0.323 | 0.334 | 0.324 |
|  | 0.414 | 0.417 | 0.411 | 0.398 |  | 0.411 | 0.457 | 0.459 | 0.488 |
|  | 0.491 | 0.499 | 0.476 | 0.493 |  | 0.543 | 0.548 | 0.533 | 0.496 |
|  | 0.527 | 0.530 | 0.434 | 0.511 |  | 0.471 | 0.492 | 0.470 | 0.437 |
|  | 0.450 | 0.437 | 0.428 | 0.443 |  | 0.514 | 0.552 | 0.545 | 0.521 |
|  | 0.471 | 0.501 | 0.476 | 0.464 |  | 0.481 | 0.493 | 0.478 | 0.474 |
|  | 0.455 | 0.518 | 0.484 | 0.484 |  | 0.522 | 0.524 | 0.540 | 0.541 |
|  | 0.416 | 0.392 | 0.400 | 0.401 |  | 0.565 | 0.501 | 0.523 | 0.507 |
|  | 0.482 | 0.502 | 0.531 | 0.501 |  | 0.425 | 0.406 | 0.401 | 0.398 |
| **Avg** | **0.448** | **0.450** | **0.437** | **0.437** |  | 0.516 | 0.548 | 0.586 | 0.602 |
| **St.Dev** | **0.042** | **0.059** | **0.051** | **0.057** |  | 0.468 | 0.466 | 0.471 | 0.496 |
|  | | | | |  | 0.516 | 0.522 | 0.558 | 0.551 |
|  |  |  |  |  |  | 0.405 | 0.412 | 0.399 | 0.388 |
|  |  |  |  |  |  | 0.407 | 0.395 | 0.419 | 0.404 |
|  |  |  |  |  |  | 0.473 | 0.523 | 0.504 | 0.482 |
|  |  |  |  |  |  | 0.482 | 0.504 | 0.486 | 0.509 |
|  |  |  |  |  |  | 0.488 | 0.501 | 0.493 | 0.512 |
|  |  |  |  |  |  | 0.462 | 0.460 | 0.443 | 0.451 |
|  |  |  |  |  |  | 0.487 | 0.491 | 0.530 | 0.519 |
|  |  |  |  |  |  | 0.457 | 0.480 | 0.483 | 0.495 |
|  |  |  |  |  |  | 0.365 | 0.379 | 0.358 | 0.388 |
|  |  |  |  |  |  | 0.590 | 0.582 | 0.555 | 0.557 |
|  |  |  |  |  |  | 0.396 | 0.436 | 0.438 | 0.437 |
|  |  |  |  |  |  | 0.398 | 0.427 | 0.395 | 0.393 |
|  |  |  |  |  |  | 0.458 | 0.469 | 0.423 | 0.437 |
|  |  |  |  |  |  | 0.579 | 0.598 | 0.559 | 0.523 |
|  |  |  |  |  |  | 0.534 | 0.517 | 0.503 | 0.515 |
|  |  |  |  |  | **Avg** | **0.460** | **0.470** | **0.465** | **0.465** |
|  |  |  |  |  | **St.Dev** | **0.073** | **0.071** | **0.073** | **0.069** |

**TMD (g/cm^3^)**

|  | **8 weeks** | **12 weeks** | **16 weeks** | **20 weeks** |  | **8 weeks** | **12 weeks** | **16 weeks** | **20 weeks** |
| --- | --- | --- | --- | --- | --- | --- | --- | --- | --- |
| **Placebo** | 1.084 | 1.121 | 1.158 | 1.175 | **LPS** | 1.119 | 1.161 | 1.193 | 1.194 |
|  | 1.086 | 1.158 | 1.171 | 1.142 |  | 1.133 | 1.188 | 1.210 | 1.215 |
|  | 1.116 | 1.179 | 1.186 | 1.232 |  | 1.081 | 1.149 | 1.179 | 1.208 |
|  | 1.040 | 1.077 | 1.111 | 1.224 |  | 1.115 | 1.186 | 1.228 | 1.196 |
|  | 1.059 | 1.086 | 1.116 | 1.212 |  | 1.074 | 1.103 | 1.112 | 1.229 |
|  | 1.032 | 1.105 | 1.105 | 1.219 |  | 1.085 | 1.116 | 1.139 | 1.244 |
|  | 1.053 | 1.074 | 1.120 | 1.216 |  | 1.090 | 1.136 | 1.144 | 1.250 |
|  | 1.097 | 1.123 | 1.138 | 1.238 |  | 1.070 | 1.099 | 1.103 | 1.200 |
|  | 1.064 | 1.086 | 1.087 | 1.219 |  | 1.079 | 1.113 | 1.138 | 1.244 |
|  | 1.089 | 1.116 | 1.126 | 1.234 |  | 0.991 | 1.095 | 1.111 | 1.226 |
|  | 1.062 | 1.117 | 1.125 | 1.222 |  | 1.082 | 1.150 | 1.172 | 1.166 |
|  | 1.095 | 1.108 | 1.115 | 1.249 |  | 1.104 | 1.166 | 1.179 | 1.166 |
| **Avg** | **1.073** | **1.112** | **1.130** | **1.215** |  | 1.123 | 1.167 | 1.201 | 1.230 |
| **St.Dev** | **0.025** | **0.032** | **0.029** | **0.029** |  | 1.094 | 1.163 | 1.164 | 1.169 |
|  | | | | |  | 1.046 | 1.064 | 1.083 | 1.182 |
|  |  |  |  |  |  | 1.067 | 1.088 | 1.109 | 1.223 |
|  |  |  |  |  |  | 1.087 | 1.115 | 1.140 | 1.244 |
|  |  |  |  |  |  | 1.091 | 1.102 | 1.117 | 1.232 |
|  |  |  |  |  |  | 1.066 | 1.096 | 1.064 | 1.248 |
|  |  |  |  |  |  | 1.049 | 1.112 | 1.092 | 1.226 |
|  |  |  |  |  |  | 1.096 | 1.134 | 1.144 | 1.164 |
|  |  |  |  |  |  | 1.106 | 1.145 | 1.170 | 1.178 |
|  |  |  |  |  |  | 1.084 | 1.168 | 1.181 | 1.184 |
|  |  |  |  |  |  | 1.103 | 1.154 | 1.187 | 1.166 |
|  |  |  |  |  |  | 1.045 | 1.052 | 1.079 | 1.190 |
|  |  |  |  |  |  | 1.075 | 1.081 | 1.088 | 1.212 |
|  |  |  |  |  |  | 1.073 | 1.111 | 1.124 | 1.241 |
|  |  |  |  |  |  | 1.072 | 1.100 | 1.137 | 1.243 |
|  |  |  |  |  |  | 1.073 | 1.122 | 1.127 | 1.249 |
|  |  |  |  |  |  | 1.064 | 1.113 | 1.080 | 1.230 |
|  |  |  |  |  | **Avg** | **1.081** | **1.125** | **1.140** | **1.212** |
|  |  |  |  |  | **St.Dev** | **0.028** | **0.035** | **0.043** | **0.030** |

**Females**

**Ct.Ar/T.Ar (%)**

|  | **8 weeks** | **12 weeks** | **16 weeks** | **20 weeks** |  | **8 weeks** | **12 weeks** | **16 weeks** | **20 weeks** |
| --- | --- | --- | --- | --- | --- | --- | --- | --- | --- |
| **Placebo** | 62.15 | 64.61 | 67.77 | 68.63 | **LPS** | 68.38 | 70.39 | 72.82 | 73.50 |
|  | 73.60 | 76.42 | 80.11 | 80.60 |  | 59.81 | 60.21 | 60.50 | 60.26 |
|  | 67.12 | 68.51 | 70.88 | 71.04 |  | 68.28 | 65.38 | 68.55 | 67.13 |
|  | 65.86 | 66.74 | 67.15 | 67.01 |  | 64.61 | 65.64 | 66.31 | 67.45 |
|  | 66.02 | 69.19 | 72.06 | 71.25 |  | 63.78 | 66.90 | 69.99 | 68.73 |
|  | 63.96 | 64.88 | 65.96 | 64.88 |  | 65.41 | 67.90 | 69.16 | 68.59 |
|  | 66.92 | 69.64 | 71.58 | 71.70 |  | 66.29 | 71.20 | 72.42 | 72.42 |
|  | 66.16 | 68.10 | 69.09 | 68.77 |  | 65.20 | 67.29 | 69.32 | 67.56 |
|  | 63.08 | 65.00 | 67.72 | 67.33 |  | 70.13 | 69.16 | 70.76 | 70.56 |
|  | 63.35 | 65.34 | 67.04 | 66.47 |  | 67.46 | 66.85 | 70.69 | 70.45 |
|  | 68.58 | 68.81 | 69.60 | 68.95 |  | 72.32 | 73.65 | 76.48 | 75.90 |
| **Avg** | **66.07** | **67.93** | **69.90** | **69.69** |  | 66.16 | 68.19 | 69.29 | 69.97 |
| **St.Dev** | **3.18** | **3.39** | **3.92** | **4.20** |  | 64.45 | 66.72 | 69.90 | 69.56 |
|  | | | | |  | 62.61 | 63.54 | 65.18 | 65.04 |
|  |  |  |  |  |  | 63.12 | 63.82 | 65.30 | 66.36 |
|  |  |  |  |  |  | 67.57 | 68.80 | 71.73 | 72.69 |
|  |  |  |  |  |  | 72.47 | 72.10 | 74.19 | 74.88 |
|  |  |  |  |  |  | 64.58 | 65.60 | 68.99 | 69.70 |
|  |  |  |  |  |  | 63.89 | 65.26 | 65.83 | 68.23 |
|  |  |  |  |  |  | 68.36 | 69.43 | 71.75 | 72.95 |
|  |  |  |  |  |  | 67.55 | 67.70 | 68.68 | 67.96 |
|  |  |  |  |  |  | 61.00 | 60.20 | 60.28 | 60.15 |
|  |  |  |  |  |  | 72.75 | 69.99 | 71.50 | 69.82 |
|  |  |  |  |  |  | 62.95 | 64.26 | 66.76 | 66.10 |
|  |  |  |  |  |  | 64.25 | 64.64 | 68.66 | 70.08 |
|  |  |  |  |  |  | 68.05 | 72.17 | 73.48 | 71.48 |
|  |  |  |  |  |  | 69.25 | 70.05 | 71.84 | 71.74 |
|  |  |  |  |  | **Avg** | **66.32** | **67.30** | **69.27** | **69.23** |
|  |  |  |  |  | **St.Dev** | **3.35** | **3.37** | **3.74** | **3.72** |

**Ct.Th (mm)**

|  | **8 weeks** | **12 weeks** | **16 weeks** | **20 weeks** |  | **8 weeks** | **12 weeks** | **16 weeks** | **20 weeks** |
| --- | --- | --- | --- | --- | --- | --- | --- | --- | --- |
| **Placebo** | 0.214 | 0.227 | 0.242 | 0.248 | **LPS** | 0.239 | 0.256 | 0.275 | 0.275 |
|  | 0.257 | 0.264 | 0.283 | 0.292 |  | 0.207 | 0.213 | 0.217 | 0.215 |
|  | 0.241 | 0.248 | 0.268 | 0.270 |  | 0.240 | 0.232 | 0.250 | 0.242 |
|  | 0.235 | 0.238 | 0.239 | 0.237 |  | 0.233 | 0.247 | 0.255 | 0.262 |
|  | 0.229 | 0.245 | 0.259 | 0.254 |  | 0.219 | 0.238 | 0.249 | 0.244 |
|  | 0.221 | 0.227 | 0.236 | 0.231 |  | 0.254 | 0.273 | 0.279 | 0.277 |
|  | 0.233 | 0.250 | 0.262 | 0.268 |  | 0.234 | 0.262 | 0.273 | 0.272 |
|  | 0.228 | 0.241 | 0.248 | 0.242 |  | 0.243 | 0.257 | 0.270 | 0.256 |
|  | 0.213 | 0.223 | 0.240 | 0.238 |  | 0.241 | 0.248 | 0.258 | 0.256 |
|  | 0.221 | 0.232 | 0.239 | 0.236 |  | 0.237 | 0.235 | 0.253 | 0.254 |
|  | 0.252 | 0.253 | 0.253 | 0.250 |  | 0.249 | 0.261 | 0.282 | 0.278 |
| **Avg** | 0.231 | 0.241 | 0.252 | 0.251 |  | 0.229 | 0.237 | 0.241 | 0.250 |
| **St.Dev** | 0.014 | 0.013 | 0.015 | 0.019 |  | 0.234 | 0.241 | 0.253 | 0.249 |
|  | | | | |  | 0.216 | 0.231 | 0.238 | 0.242 |
|  |  |  |  |  |  | 0.212 | 0.223 | 0.223 | 0.228 |
|  |  |  |  |  |  | 0.229 | 0.241 | 0.252 | 0.258 |
|  |  |  |  |  |  | 0.253 | 0.252 | 0.263 | 0.264 |
|  |  |  |  |  |  | 0.229 | 0.235 | 0.251 | 0.254 |
|  |  |  |  |  |  | 0.222 | 0.224 | 0.240 | 0.250 |
|  |  |  |  |  |  | 0.235 | 0.246 | 0.256 | 0.260 |
|  |  |  |  |  |  | 0.229 | 0.232 | 0.243 | 0.239 |
|  |  |  |  |  |  | 0.226 | 0.228 | 0.230 | 0.230 |
|  |  |  |  |  |  | 0.263 | 0.245 | 0.245 | 0.239 |
|  |  |  |  |  |  | 0.218 | 0.223 | 0.237 | 0.234 |
|  |  |  |  |  |  | 0.227 | 0.232 | 0.255 | 0.260 |
|  |  |  |  |  |  | 0.235 | 0.258 | 0.269 | 0.261 |
|  |  |  |  |  |  | 0.236 | 0.244 | 0.257 | 0.256 |
|  |  |  |  |  | **Avg** | **0.233** | **0.241** | **0.252** | **0.252** |
|  |  |  |  |  | **St.Dev** | **0.013** | **0.014** | **0.016** | **0.015** |

**Ps.Pm (mm)**

|  | **8 weeks** | **12 weeks** | **16 weeks** | **20 weeks** |  | **8 weeks** | **12 weeks** | **16 weeks** | **20 weeks** |
| --- | --- | --- | --- | --- | --- | --- | --- | --- | --- |
| **Placebo** | 6.764 | 6.729 | 6.697 | 6.725 | **LPS** | 6.731 | 6.939 | 7.001 | 6.951 |
|  | 6.174 | 5.916 | 5.757 | 5.876 |  | 7.258 | 7.398 | 7.655 | 7.706 |
|  | 7.040 | 7.003 | 7.173 | 7.200 |  | 6.358 | 6.759 | 6.857 | 6.915 |
|  | 6.718 | 6.748 | 6.810 | 6.819 |  | 6.938 | 7.395 | 7.612 | 7.712 |
|  | 6.736 | 6.586 | 6.450 | 6.512 |  | 6.940 | 6.979 | 7.014 | 7.083 |
|  | 7.083 | 7.186 | 7.172 | 7.243 |  | 7.236 | 7.410 | 7.640 | 7.812 |
|  | 6.576 | 6.588 | 6.574 | 6.828 |  | 6.583 | 6.569 | 6.706 | 6.723 |
|  | 6.802 | 6.750 | 6.797 | 6.849 |  | 7.441 | 7.554 | 7.391 | 7.425 |
|  | 6.845 | 6.830 | 6.945 | 7.000 |  | 6.313 | 6.962 | 7.001 | 6.954 |
|  | 6.949 | 6.939 | 6.882 | 6.961 |  | 6.628 | 6.723 | 6.700 | 6.736 |
|  | 6.694 | 6.690 | 6.672 | 6.717 |  | 6.151 | 6.286 | 6.186 | 6.307 |
| **Avg** | 6.762 | 6.724 | 6.721 | 6.794 |  | 6.749 | 6.673 | 6.683 | 6.872 |
| **St.Dev** | 0.247 | 0.323 | 0.390 | 0.371 |  | 6.890 | 6.905 | 6.842 | 6.951 |
|  | | | | |  | 6.854 | 7.303 | 7.318 | 7.378 |
|  |  |  |  |  |  | 6.980 | 7.114 | 6.954 | 6.937 |
|  |  |  |  |  |  | 6.433 | 6.441 | 6.495 | 6.573 |
|  |  |  |  |  |  | 6.285 | 6.529 | 6.614 | 6.389 |
|  |  |  |  |  |  | 6.893 | 6.968 | 6.943 | 7.069 |
|  |  |  |  |  |  | 6.957 | 7.000 | 7.379 | 7.285 |
|  |  |  |  |  |  | 6.412 | 6.628 | 6.659 | 6.663 |
|  |  |  |  |  |  | 6.601 | 6.659 | 6.732 | 6.855 |
|  |  |  |  |  |  | 7.747 | 8.047 | 8.197 | 8.373 |
|  |  |  |  |  |  | 6.679 | 6.850 | 6.648 | 6.718 |
|  |  |  |  |  |  | 6.881 | 6.676 | 6.803 | 6.896 |
|  |  |  |  |  |  | 6.709 | 6.914 | 6.830 | 6.815 |
|  |  |  |  |  |  | 6.671 | 6.550 | 6.886 | 7.127 |
|  |  |  |  |  |  | 6.425 | 6.377 | 6.469 | 6.522 |
|  |  |  |  |  | **Avg** | **6.768** | **6.911** | **6.971** | **7.028** |
|  |  |  |  |  | **St.Dev** | **0.367** | **0.404** | **0.442** | **0.467** |

**Ec.Pm (mm)**

|  | **8 weeks** | **12 weeks** | **16 weeks** | **20 weeks** |  | **8 weeks** | **12 weeks** | **16 weeks** | **20 weeks** |
| --- | --- | --- | --- | --- | --- | --- | --- | --- | --- |
| **Placebo** | 2.639 | 2.538 | 2.461 | 2.431 | **LPS** | 2.487 | 2.511 | 2.401 | 2.349 |
|  | 2.095 | 1.928 | 1.753 | 1.761 |  | 2.856 | 2.880 | 2.982 | 3.038 |
|  | 2.668 | 2.591 | 2.562 | 2.560 |  | 2.290 | 2.477 | 2.430 | 2.496 |
|  | 2.477 | 2.471 | 2.470 | 2.482 |  | 2.570 | 2.694 | 2.750 | 2.745 |
|  | 2.561 | 2.396 | 2.258 | 2.312 |  | 2.709 | 2.615 | 2.538 | 2.598 |
|  | 2.763 | 2.742 | 2.665 | 2.725 |  | 2.667 | 2.664 | 2.682 | 2.761 |
|  | 2.488 | 2.366 | 2.294 | 2.401 |  | 2.429 | 2.293 | 2.289 | 2.282 |
|  | 2.612 | 2.504 | 2.457 | 2.532 |  | 2.864 | 2.789 | 2.643 | 2.699 |
|  | 2.704 | 2.649 | 2.604 | 2.632 |  | 2.266 | 2.494 | 2.436 | 2.459 |
|  | 2.684 | 2.658 | 2.553 | 2.610 |  | 2.414 | 2.464 | 2.327 | 2.338 |
|  | 2.401 | 2.383 | 2.371 | 2.398 |  | 2.204 | 2.202 | 2.031 | 2.111 |
| **Avg** | 2.554 | 2.475 | 2.404 | 2.440 |  | 2.497 | 2.397 | 2.359 | 2.413 |
| **St.Dev** | 0.187 | 0.219 | 0.250 | 0.255 |  | 2.578 | 2.506 | 2.392 | 2.426 |
|  | | | | |  | 2.654 | 2.797 | 2.770 | 2.756 |
|  |  |  |  |  |  | 2.707 | 2.722 | 2.612 | 2.587 |
|  |  |  |  |  |  | 2.418 | 2.357 | 2.295 | 2.300 |
|  |  |  |  |  |  | 2.231 | 2.348 | 2.260 | 2.180 |
|  |  |  |  |  |  | 2.611 | 2.591 | 2.477 | 2.486 |
|  |  |  |  |  |  | 2.668 | 2.677 | 2.758 | 2.673 |
|  |  |  |  |  |  | 2.281 | 2.320 | 2.250 | 2.209 |
|  |  |  |  |  |  | 2.514 | 2.521 | 2.474 | 2.539 |
|  |  |  |  |  |  | 3.081 | 3.187 | 3.214 | 3.294 |
|  |  |  |  |  |  | 2.316 | 2.521 | 2.401 | 2.452 |
|  |  |  |  |  |  | 2.655 | 2.519 | 2.507 | 2.559 |
|  |  |  |  |  |  | 2.558 | 2.649 | 2.474 | 2.465 |
|  |  |  |  |  |  | 2.436 | 2.261 | 2.304 | 2.425 |
|  |  |  |  |  |  | 2.373 | 2.285 | 2.252 | 2.286 |
|  |  |  |  |  | **Avg** | **2.531** | **2.546** | **2.493** | **2.516** |
|  |  |  |  |  | **St.Dev** | **0.212** | **0.219** | **0.250** | **0.259** |

**Tt.Ar(mm^2^)**

|  | **8 weeks** | **12 weeks** | **16 weeks** | **20 weeks** |  | **8 weeks** | **12 weeks** | **16 weeks** | **20 weeks** |
| --- | --- | --- | --- | --- | --- | --- | --- | --- | --- |
| **Placebo** | 1.165 | 1.180 | 1.196 | 1.214 | **LPS** | 1.175 | 1.263 | 1.323 | 1.301 |
|  | 1.079 | 1.020 | 1.017 | 1.065 |  | 1.257 | 1.308 | 1.371 | 1.376 |
|  | 1.262 | 1.267 | 1.356 | 1.370 |  | 1.119 | 1.198 | 1.251 | 1.245 |
|  | 1.198 | 1.203 | 1.213 | 1.206 |  | 1.249 | 1.394 | 1.465 | 1.498 |
|  | 1.170 | 1.166 | 1.158 | 1.163 |  | 1.189 | 1.241 | 1.250 | 1.257 |
|  | 1.221 | 1.257 | 1.285 | 1.287 |  | 1.407 | 1.489 | 1.542 | 1.579 |
|  | 1.145 | 1.181 | 1.205 | 1.275 |  | 1.163 | 1.210 | 1.265 | 1.262 |
|  | 1.173 | 1.194 | 1.219 | 1.205 |  | 1.388 | 1.443 | 1.440 | 1.405 |
|  | 1.158 | 1.172 | 1.230 | 1.239 |  | 1.083 | 1.246 | 1.276 | 1.262 |
|  | 1.215 | 1.231 | 1.228 | 1.235 |  | 1.163 | 1.181 | 1.200 | 1.212 |
|  | 1.231 | 1.228 | 1.215 | 1.217 |  | 1.059 | 1.112 | 1.140 | 1.153 |
| **Avg** | 1.183 | 1.191 | 1.211 | 1.225 |  | 1.168 | 1.159 | 1.162 | 1.229 |
| **St.Dev** | 0.049 | 0.066 | 0.082 | 0.076 |  | 1.249 | 1.249 | 1.240 | 1.246 |
|  | | | | |  | 1.181 | 1.327 | 1.333 | 1.372 |
|  |  |  |  |  |  | 1.170 | 1.244 | 1.189 | 1.193 |
|  |  |  |  |  |  | 1.088 | 1.130 | 1.139 | 1.168 |
|  |  |  |  |  |  | 1.095 | 1.140 | 1.173 | 1.126 |
|  |  |  |  |  |  | 1.223 | 1.246 | 1.262 | 1.290 |
|  |  |  |  |  |  | 1.211 | 1.203 | 1.343 | 1.335 |
|  |  |  |  |  |  | 1.102 | 1.174 | 1.187 | 1.188 |
|  |  |  |  |  |  | 1.121 | 1.143 | 1.192 | 1.207 |
|  |  |  |  |  |  | 1.433 | 1.522 | 1.566 | 1.604 |
|  |  |  |  |  |  | 1.209 | 1.200 | 1.138 | 1.152 |
|  |  |  |  |  |  | 1.190 | 1.157 | 1.209 | 1.222 |
|  |  |  |  |  |  | 1.184 | 1.243 | 1.267 | 1.264 |
|  |  |  |  |  |  | 1.152 | 1.169 | 1.260 | 1.303 |
|  |  |  |  |  |  | 1.096 | 1.113 | 1.158 | 1.166 |
|  |  |  |  |  | **Avg** | **1.190** | **1.241** | **1.272** | **1.282** |
|  |  |  |  |  | **St.Dev** | **0.096** | **0.110** | **0.119** | **0.124** |

**Ma.Ar (mm^2^)**

|  | **8 weeks** | **12 weeks** | **16 weeks** | **20 weeks** |  | **8 weeks** | **12 weeks** | **16 weeks** | **20 weeks** |
| --- | --- | --- | --- | --- | --- | --- | --- | --- | --- |
| **Placebo** | 0.441 | 0.418 | 0.385 | 0.381 | **LPS** | 0.372 | 0.374 | 0.360 | 0.345 |
|  | 0.285 | 0.240 | 0.202 | 0.207 |  | 0.505 | 0.520 | 0.542 | 0.547 |
|  | 0.415 | 0.399 | 0.395 | 0.397 |  | 0.355 | 0.415 | 0.394 | 0.409 |
|  | 0.409 | 0.400 | 0.399 | 0.398 |  | 0.442 | 0.479 | 0.494 | 0.488 |
|  | 0.398 | 0.359 | 0.324 | 0.334 |  | 0.431 | 0.411 | 0.375 | 0.393 |
|  | 0.440 | 0.442 | 0.437 | 0.452 |  | 0.487 | 0.478 | 0.476 | 0.496 |
|  | 0.379 | 0.358 | 0.342 | 0.361 |  | 0.392 | 0.348 | 0.349 | 0.348 |
|  | 0.397 | 0.381 | 0.377 | 0.376 |  | 0.483 | 0.472 | 0.442 | 0.456 |
|  | 0.428 | 0.410 | 0.397 | 0.405 |  | 0.324 | 0.384 | 0.373 | 0.372 |
|  | 0.445 | 0.427 | 0.405 | 0.414 |  | 0.379 | 0.391 | 0.352 | 0.358 |
|  | 0.387 | 0.383 | 0.369 | 0.378 |  | 0.293 | 0.293 | 0.268 | 0.278 |
| **Avg** | 0.402 | 0.383 | 0.367 | 0.373 |  | 0.395 | 0.369 | 0.357 | 0.369 |
| **St.Dev** | 0.045 | 0.054 | 0.063 | 0.063 |  | 0.444 | 0.416 | 0.373 | 0.379 |
|  | | | | |  | 0.442 | 0.484 | 0.464 | 0.480 |
|  |  |  |  |  |  | 0.431 | 0.450 | 0.413 | 0.401 |
|  |  |  |  |  |  | 0.353 | 0.352 | 0.322 | 0.319 |
|  |  |  |  |  |  | 0.301 | 0.318 | 0.303 | 0.283 |
|  |  |  |  |  |  | 0.433 | 0.429 | 0.391 | 0.391 |
|  |  |  |  |  |  | 0.437 | 0.418 | 0.459 | 0.424 |
|  |  |  |  |  |  | 0.349 | 0.359 | 0.335 | 0.321 |
|  |  |  |  |  |  | 0.364 | 0.369 | 0.373 | 0.387 |
|  |  |  |  |  |  | 0.559 | 0.606 | 0.622 | 0.639 |
|  |  |  |  |  |  | 0.330 | 0.360 | 0.324 | 0.348 |
|  |  |  |  |  |  | 0.441 | 0.414 | 0.402 | 0.414 |
|  |  |  |  |  |  | 0.423 | 0.440 | 0.397 | 0.378 |
|  |  |  |  |  |  | 0.368 | 0.325 | 0.334 | 0.372 |
|  |  |  |  |  |  | 0.337 | 0.333 | 0.326 | 0.329 |
|  |  |  |  |  | **Avg** | **0.403** | **0.408** | **0.393** | **0.397** |
|  |  |  |  |  | **St.Dev** | **0.065** | **0.069** | **0.077** | **0.080** |

**TMD (g/cm^3^)**

|  | **8 weeks** | **12 weeks** | **16 weeks** | **20 weeks** |  | **8 weeks** | **12 weeks** | **16 weeks** | **20 weeks** |
| --- | --- | --- | --- | --- | --- | --- | --- | --- | --- |
| **Placebo** | 1.116 | 1.178 | 1.220 | 1.224 | **LPS** | 1.158 | 1.200 | 1.239 | 1.260 |
|  | 1.166 | 1.228 | 1.273 | 1.303 |  | 1.116 | 1.160 | 1.170 | 1.205 |
|  | 1.162 | 1.207 | 1.236 | 1.261 |  | 1.177 | 1.190 | 1.224 | 1.242 |
|  | 1.170 | 1.193 | 1.217 | 1.237 |  | 1.161 | 1.206 | 1.229 | 1.257 |
|  | 1.172 | 1.222 | 1.248 | 1.263 |  | 1.149 | 1.184 | 1.221 | 1.241 |
|  | 1.161 | 1.187 | 1.186 | 1.205 |  | 1.177 | 1.227 | 1.249 | 1.260 |
|  | 1.172 | 1.192 | 1.194 | 1.275 |  | 1.162 | 1.219 | 1.244 | 1.270 |
|  | 1.138 | 1.160 | 1.177 | 1.206 |  | 1.153 | 1.167 | 1.219 | 1.258 |
|  | 1.109 | 1.151 | 1.191 | 1.202 |  | 1.146 | 1.157 | 1.220 | 1.236 |
|  | 1.145 | 1.191 | 1.205 | 1.246 |  | 1.132 | 1.182 | 1.222 | 1.225 |
|  | 1.192 | 1.222 | 1.232 | 1.282 |  | 1.180 | 1.222 | 1.247 | 1.256 |
| **Avg** | 1.155 | 1.194 | 1.216 | 1.246 |  | 1.162 | 1.209 | 1.240 | 1.246 |
| **St.Dev** | 0.025 | 0.025 | 0.029 | 0.034 |  | 1.167 | 1.196 | 1.243 | 1.263 |
|  | | | | |  | 1.152 | 1.183 | 1.241 | 1.266 |
|  |  |  |  |  |  | 1.159 | 1.179 | 1.197 | 1.223 |
|  |  |  |  |  |  | 1.147 | 1.194 | 1.253 | 1.282 |
|  |  |  |  |  |  | 1.145 | 1.202 | 1.227 | 1.243 |
|  |  |  |  |  |  | 1.163 | 1.202 | 1.252 | 1.270 |
|  |  |  |  |  |  | 1.132 | 1.185 | 1.201 | 1.239 |
|  |  |  |  |  |  | 1.178 | 1.197 | 1.222 | 1.254 |
|  |  |  |  |  |  | 1.153 | 1.197 | 1.208 | 1.246 |
|  |  |  |  |  |  | 1.155 | 1.192 | 1.178 | 1.225 |
|  |  |  |  |  |  | 1.171 | 1.201 | 1.227 | 1.230 |
|  |  |  |  |  |  | 1.142 | 1.191 | 1.222 | 1.237 |
|  |  |  |  |  |  | 1.112 | 1.172 | 1.209 | 1.244 |
|  |  |  |  |  |  | 1.164 | 1.228 | 1.255 | 1.257 |
|  |  |  |  |  |  | 1.186 | 1.223 | 1.218 | 1.268 |
|  |  |  |  |  | **Avg** | **1.156** | **1.195** | **1.225** | **1.248** |
|  |  |  |  |  | **St.Dev** | **0.018** | **0.019** | **0.022** | **0.018** |
